# Correction of Scanning Electron Microscope Imaging Artifacts in a Novel Digital Image Correlation Framework

**DOI:** 10.1007/s11340-018-00469-w

**Published:** 2019-03-12

**Authors:** S. Maraghechi, J. P. M. Hoefnagels, R. H. J. Peerlings, O. Rokoš, M. G. D. Geers

**Affiliations:** Department of Mechanical Engineering, Eindhoven University of Technology, 5600MB Eindhoven, The Netherlands

**Keywords:** Integrated digital image correlation, Scanning electron microscopy, Imaging artifacts, Spatial distortion, Drift distortion, Line shift artifacts

## Abstract

The combination of digital image correlation (DIC) and scanning electron microscopy (SEM) enables to extract high resolution full field displacement data, based on the high spatial resolution of SEM and the sub-pixel accuracy of DIC. However, SEM images may exhibit a considerable amount of imaging artifacts, which may seriously compromise the accuracy of the displacements and strains measured from these images. The current study proposes a unified general framework to correct for the three dominant types of SEM artifacts, i.e. spatial distortion, drift distortion and scan line shifts. The artifact fields are measured alongside the mechanical deformations to minimize the artifact induced errors in the latter. To this purpose, Integrated DIC (IDIC) is extended with a series of hierarchical mapping functions that describe the interaction of the imaging process with the mechanics. A new IDIC formulation based on these mapping functions is derived and the potential of the framework is tested by a number of virtual experiments. The effect of noise in the images and different regularization options for the artifact fields are studied. The error in the mechanical displacement fields measured for noise levels up to 5*%* is within the usual DIC accuracy range for all the cases studied, while it is more than 4 pixels if artifacts are ignored. A validation on real SEM images at three different magnifications confirms that all three distortion fields are accurately captured. The results of all virtual and real experiments demonstrate the accuracy of the methodology proposed, as well as its robustness in terms of convergence.

## Introduction

Digital Image Correlation (DIC) is nowadays the most frequently used full-field displacement measurement technique for industrial and academic purposes [1]. Apart from conventional optical images (taken with one or multiple cameras), DIC may be applied to microscopy images obtained by different methods, such as scanning electron microscopy [2], scanning tunneling microscopy [3], atomic force microscopy [4, 5], high-resolution transmission electron microscopy [3, 6], and optical profilometry [7]. These methods provide a high spatial resolution which, combined with the sub-pixel accuracy of DIC [1], enable a high resolution displacement and strain assessment. This opens a vast perspective in experimental micromechanics. Scanning electron microscopy (SEM) has proven itself to be one of the most powerful microscopy methods available. It combines a high spatial resolution (e.g. with respect to light microscopy or optical profilometry) with relative ease of use (e.g. with respect to transmission electron microscopy or atomic force microscopy). However, using SEM images for kinematic measurements comes with a price, due to the presence of several complicated imaging artifacts [8, 9]. These artifacts manifest themselves in the form of distortions in the image, and cause significant artificial deformations and strains in DIC measurements if ignored [10, 11].

SEM imaging artifacts can be categorized into three classes according to Ref. [12]. (1) Random, time-dependent distortion due to positioning errors of the electron beam during scanning, referred to as “scan line shifts”. (2) Non-random, time-independent spatial distortion, similar to distortions observed in optical systems. (3) Non-random, time-dependent distortion referred to as drift distortion. It triggers non-uniform artificial deformation fields in images and directly results from the scanning involved in the SEM imaging process [13, 14].

The effect of the above-mentioned artifacts may be reduced by optimizing the SEM scanning parameters. For instance, faster scanning can reduce drift distortions, while lower beam voltage and smaller spot size may reduce charging leading to less drift distortions. However, these alterations do not eliminate the artifacts, while they increase the image noise, which may also reduce the accuracy of the mechanical deformation field to be identified.

In this paper, a novel framework for correcting all three categories of SEM imaging artifacts is presented in a unified and systematic way. The method is fully integrated in a DIC framework and thus yields, at the same time, artifact-corrected images and accurate mechanical deformation fields.

The different types of artifacts have been studied in the literature and some solutions have been proposed to correct them. Several methods have been proposed to correct the drift artifact in SEM images [4, 14–25]. Most of these papers focus only on drift distortion at high magnification, where the effect of drift distortion is more significant than that of spatial distortion. The spatial distortion is discussed in other papers and different solutions are also proposed for correcting it. These studies cover optical microscopy [12, 26, 27] as well as SEM [11, 24, 28, 29]. The random, time-dependent scan line shift artifact has been studied much less and is often neglected in the literature. Lagattu et al. [30] and Stinville et al. [31] report on the presence of this artifact in SEM images and Sutton et al. [10] proposes averaging over a number of images to reduce the detrimental effect of the line shifts. In a previous study by the authors, a more rigorous solution was proposed, based on the enrichment of conventional Global Digital Image Correlation (GDIC) basis functions by error functions; this method was demonstrated to be effective in quantifying the line shifts with amplitudes ranging between 0.5 − 5 px, and correcting them to less than 0.01 px error [32].

Still lacking in the literature is a systematic unified framework to simultaneously quantify all three types of SEM imaging artifacts along with the mechanical displacement field in an integrated general solution scheme. The methodology introduced by Sutton et al. [11] can be considered ground-breaking, in the sense that, by correcting the DIC displacements it simultaneously deals with spatial and drift distortion and decreases the effect of line shifts (effective for line shifts with amplitudes of up to 1 pixel), but it requires averaging of multiple (as many as 16) scans, and involves many separate optimization steps^1^ for characterizing drift and spatial distortion properly. Simultaneously addressing all three artifact types is indeed quite challenging, since one can easily render the methodology ill-posed and non-unique.

The objective of the current study is therefore to fill this gap, and to develop a systematic, stable and unified method to correct for all three types of SEM artifacts in a generic DIC framework.

The three types of SEM artifacts discussed above show a deterministic behavior. Based on this fact, in the current study, Integrated Digital Image Correlation (IDIC) is used to measure these artifact fields alongside the mechanical displacements in a separate manner. Such a measurement will ensure that the artifacts induce minimum errors in the strain measurements. To this end, the imaging process in the SEM as well as the mechanical deformation in the specimen are modeled as a hierarchy of mathematical mapping functions to replace the conventional mapping functions used in IDIC and GDIC. Such a composition of mapping functions enables to uniquely capture the mechanics and artifacts independently. The general framework is not restricted to SEM images and can be equally applied for correcting imaging artifacts for other microscopy methods, whereby the hierarchy of mapping functions needs to be adapted to the specific microscopy technique. Here we focus on SEM imaging and develop the method for it accordingly.

In this study, proper regularization functions are chosen to describe spatial distortion, drift distortion and scan line shifts in SEM images. (1) Line shifts are randomly occurring and hence are to be extracted from an image when and where they occur (as reported in our previous study [32]). (2) Spatial distortion is independent of time, i.e. equal for all the images. Hence, it can be captured by a calibration phase prior to the mechanical test based on a simple known mechanical field, i.e. rigid body motion [15]. (3) The drift distortion on the other hand is a time dependent phenomenon that is smooth in time [11]. Thus it is defined and regularized in time as a smooth function covering the scanning time of all the images in a mechanical test. The drift distortion as a function of time is projected on the images by the mathematical definition of the scanning process in time [11].

The DIC problem is then solved in a time-integrated manner, correlating all deformation and distortion fields of all images at once [33]. Finally, since all images (including the first one) contain distortions, the existence of an undeformed reference image must be abandoned; therefore, a more general definition of the reference image is introduced, based on the average of all back-deformed images.

The paper presents the methodology in detail, followed by a proof of principle by means of a series of virtual experiments. It will be demonstrated that this framework has several characteristic advantages, justifying the originality of the work:

(i) all artifacts are dealt with in a single unified framework,
(ii) only two correlation phases (spatial distortion calibration and mechanical test phase) suffice to assure that all artifact distortion fields are captured accurately along with the mechanical deformation field,
(iii) the information in all images is optimally used by avoiding any kind of image integration,
(iv) drift distortion is directly measured and corrected in every image, including the first (reference) image, without any extrapolation of data and,
(v) there is no need to correct the images and perform another correlation on the corrected images again; i.e. no pre- or post- processing of images or DIC displacement data is needed (Table 1).

**Table 1:** Notational conventions

Scalar: *a*	Column: $\underline {a}$
Vector: **a**, **A**	Matrix: $\underline {\underline {A}}$
Inner product of vectors: **a**.**b**	${\mathbf {\nabla }}_{\mathbf {x}} = \frac {\partial }{\partial x}\mathbf {e}_{x} + \frac {\partial }{\partial y}\mathbf {e}_{y}$
Composition of functions : $f \circ g(x) = f \left (g \left (x \right ) \right )$	

## SEM Imaging Artifacts

SEM images are generated by the interaction of an electron beam focused on the specimen surface and registering the out-coming electrons, point by point in a scanning process. The electron beam, after being generated and concentrated by a series of lenses (electromagnetic or electrostatic), follows along the optical axis up to the point where it passes through the scanning coils. Here the electron beam is deflected from the optical axis in intervals to perform the scanning of the specimen surface. A final electromagnetic lens following the scanning coils focuses the beam onto the specimen surface. The schematic representation in Fig. 1(a) depicts these successive imaging steps.

**Fig. 1:**
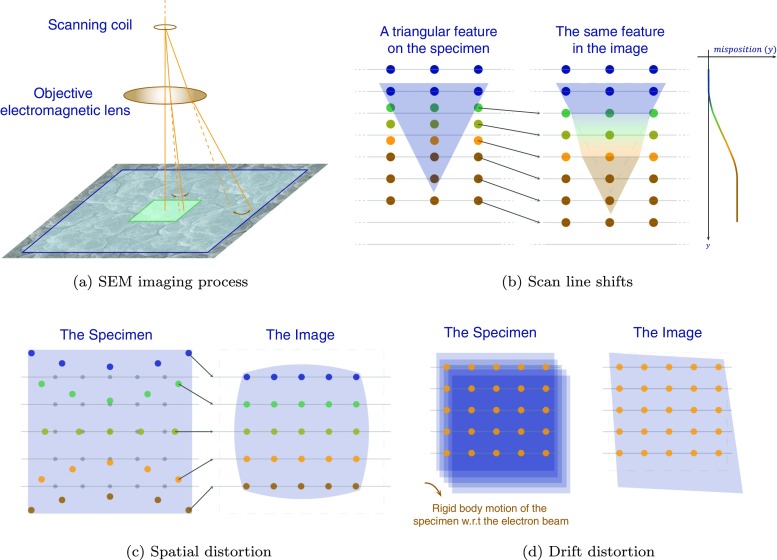
Schematic representation of the origin of SEM imaging artifacts: **a** SEM imaging process and the effect of the inhomogeneous electromagnetic field of the final electromagnetic lens in different magnifications; **b** line shifts: errors occurring in the positioning of the electron beam on the surface of the specimen are reflected as a localized shift in the image (schematic of a zoomed view of a feature on the specimen); **c** spatial distortion: the scanning occurs on the colored dots instead of the correct positions (gray dots) which is reflected in the image as the spatial distortion (schematic of the whole field of view); **d** drift distortion: undesired relative motion of the specimen with respect to the electron beam during scanning results in shear/tension like distortions in SEM images (schematic of the whole field of view). The gray horizontal lines in (b), (c) and (d) indicate the scan lines, i.e. where the scanning should have happened in case of no beam positioning error, thus the pixel positions

An error in the deflection of the electron beam in the scanning coils from one scan line to the other is considered to be the main source of scan line shifts [32]. The origin of such errors is not discussed in the literature. A speculative explanation is that, line shifts may be caused by the (sudden) discharge of spurious contamination particles on the wall of the electron column, which gradually charge up over time. Line shift artifacts occur in a random manner, however, they reveal a deterministic flaw in the image. Figure 1(b) shows how such a mis-positioning of the electron beam is reflected as a local distortion in the image.

The electron beam, which is deflected by the scanning coils to a certain pixel position possibly entailing a scan line shift, now passes through the (final) objective electromagnetic lens. The electromagnetic field of the objective lens is always spatially nonuniform to a certain extent. Thus the beam is further distorted depending on where it passes through the objective lens. In the scanning process, the further the beam passes from the center of the electromagnetic lens, the higher the deviation from the desired magnetic field that acts on the beam, i.e. the higher the erroneous (radial) deflection of the beam. This can be observed in the fact that images with lower magnification generally exhibit more spatial distortion, see Fig. 1(a). This distortion in the electron beam is assumed to be the source of the spatial distortion artifact, Fig. 1(c). These distortion fields are well studied in the literature for aberration-corrected electron microscopes [34, 35]. The spatial distortion is assumed to be a time-independent field. This means that as long as the electron beam parameters are not altered, the distortion field is equally affecting all of the images in a series [11].

The drift distortion artifact is a consequence of undesired motion of the specimen relative to the electron beam while the scanning process is going on, see Fig. 1(d). This smooth time-dependent motion can be caused, for instance, by the motion of the stage or different components of the SEM column (e.g. due to temperature changes), or by global repulsion of the beam due to charging of the specimen that increases in time [36]. Although new microscopes give vast possibilities for scanning procedures, it is common that the scanning is performed row by row, typically from top to bottom of the image, or sometimes column by column. Such a scanning scheme results in distortions which induce apparent tension/compression and shear. The drift distortion field is thus non-uniform in each image, and varies from image to image [11].

## Methodology

### Novel IDIC Formulation Based on Hierarchical Mapping Functions for SEM Artifacts

Images are considered as mathematical projections of a reference pattern. This reference pattern, *F*, is an ideal, instantaneous (thus not real) image of the specimen, free of any artifacts, at the very first instance *t* = 0 when the scanning of the first image starts. Consider a material point **X**, on a specimen at this instance, Fig. 2(a). Due to mechanical deformation and drift (the rigid body motion of the specimen with respect to the electron beam during scanning), this material point will be located in another position **x** at the moment it is scanned in a certain image. The mapping between **X** and **x** is defined as:

1 $$ {\mathbf{x}} = {{\boldsymbol{\phi}}_{M}} ({\mathbf{X}}),  $$

where subscript *M* refers to “Motion” in the plane of the specimen. This mapping function incorporates mechanical deformation and drift artifact. On the other hand, the imaging process introduces errors as well. The electron beam landed in position **x** while it was supposed to scan another position, ***ξ***. This mispositioning is described by a second mapping function that incorporates the imaging artifacts,

2 $$ {\mathbf{x}} = {{\boldsymbol{\psi}}_{I}} ({\boldsymbol{\xi}}),  $$

where the subscript *I* refers to “Imaging”. The position vector ***ξ*** indicates the position in the image plane where the gray scale data of the position **x**in the specimen is recorded, i.e. ***ξ*** is the pixel position, Fig. 2(a). Note that the mathematical formulations in this section are all in continuous form, however, the final calculations are done in discrete manner since the digital images are constructed of discrete data based on pixels. For the correlation, the pixel position ***ξ*** corresponding to each material point **X** is needed. To this end, the mapping function in equation (2) needs to be inverted:

3 $$ {\boldsymbol{\xi}} = {{\boldsymbol{\psi}}_{I}}^{-1}({\mathbf{x}}) = {{\boldsymbol{\phi}}_{I}} ({\mathbf{x}}) . $$

By combining equations (1) and (3) the total mapping from each material point to the correct pixel position is attained as:

4 $$ {\boldsymbol{\xi}} = {{\boldsymbol{\phi}}_{I}} \left( {{\boldsymbol{\phi}}_{M}} ({\mathbf{X}}) \right) , \quad \text{or} \quad {\boldsymbol{\xi}} = {{\boldsymbol{\phi}}_{I}} \circ {{\boldsymbol{\phi}}_{M}} ({\mathbf{X}}),  $$

where the symbol “∘” denotes the classical function composition. This hierarchical mapping for a certain image *g* is depicted in Fig. 2(a). Note that the pixel position, ***ξ***, is not necessarily an integer value, thus requiring interpolation in between pixels to recover the desired gray scale value.

**Fig. 2:**
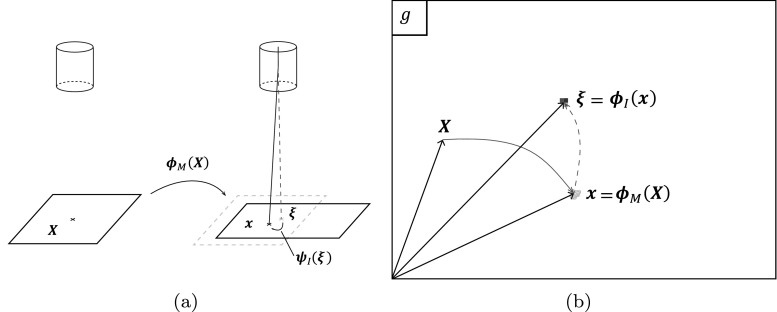
The process of imaging in the SEM and the corresponding mapping functions: **a** a material point **X** before starting the first image of a mechanical test ends in position **x** at the moment it is scanned due to mechanical deformation and drift (rigid body motion of the specimen with respect to the electron beam), and the electron beam lands in position **x** instead of ***ξ*** due to imaging artifacts. **b** Projection of the mapping functions on a certain image *g* showing the hierarchical mapping of material point **X** to the corresponding pixel position ***ξ*** in this image

#### Mapping function for imaging

Let us elaborate on ***ψ***_*I*_(***ξ***) resulting from the SEM imaging process. Based on the order discussed in the previous section, ***ψ***_*I*_ is a sequential composition of two mapping functions, the line shift mapping function, ***ψ***_*L*_, and the spatial distortion mapping function, ***ψ***_*S*_, which for image *i* results in:

5 $$ {{\boldsymbol{\psi}}_{I}}_{i}({\boldsymbol{\xi}}_{i}) = {\boldsymbol{\psi}}_{S} \circ {{\boldsymbol{\psi}}_{L}}_{i}({\boldsymbol{\xi}}_{i}).  $$

Note that ***ψ***_*S*_ is constant between images. As in equation (3), the inverse of the imaging mapping function for image *i* is:

6 $$ {\boldsymbol{\xi}}_{i} = {{\boldsymbol{\phi}}_{I}}_{i}({\mathbf{x}}_{i}) = {{\boldsymbol{\psi}}_{I}}^{-1}_{i}({\mathbf{x}}_{i}) = {{\boldsymbol{\phi}}_{L}}_{i} \circ {\boldsymbol{\phi}}_{S}({\mathbf{x}}_{i}).  $$

Note that the spatial distortion field is independent of the image, since it is assumed to be time-independent.

#### Mapping function for motion in the specimen plane

On the other hand the motion mapping function, ***ϕ***_*M*_, is defined for image *i* as:

7 $$ {{\boldsymbol{\phi}}_{M}}_{i}({\mathbf{X}},{\boldsymbol{\xi}}) = {\mathbf{X}} + {\mathbf{U}}_{i}({\mathbf{X}}) + {\mathbf{D}}(t({\boldsymbol{\xi}}))  $$

where **U**(**X**) is the mechanical displacement field and **D**(*t*) is the drift, which is the relative motion of the specimen with respect to the electron beam in time. Note that **U**_*i*_(**X**), which is applied in discrete load increments, may contain a physical rigid body motion of the specimen as well, but this applied discrete rigid body motion does not induce artificial strains, in contrast to the smooth, time-continuous rigid body motion during scanning that is caused by drift. The two fields are separated by constraining the mechanics to be equal for every pair of images (taking two images per load step) while drift distortion is a smooth function in time. This strategy is explained in more detail in “Correlation Procedure”. Since relative beam-specimen motion, triggering drift distortion, goes on during the scanning process (and during the time between the images), its value differs from pixel to pixel. Following Ref. [15], these scan times can be projected on the image plane (the pixel positions ***ξ***) based on a mathematical definition of the scanning process:

8 $$ t({\boldsymbol{\xi}}) = t_{i} + (t_{d} \mathbf{e}_{x} + t_{r} \mathbf{e}_{y}) \cdot {\boldsymbol{\xi}} \quad ; \quad t_{r} = W t_{d} + t_{j}, $$

where *t*_*d*_ is the dwell time describing the amount of time spent on the scanning of each single point resulting in a single pixel; *t*_*r*_ is the time required to scan one line while *t*_*j*_ is the time required to re-position the beam from the end of one scan line to the beginning of the next one; *t*_*i*_ is the elapsed time until the beginning of scanning of image *i*; *W* is the width of the image (length of each scan line) in pixels, and **e**_*x*_ and **e**_*y*_ are base vectors in *x* and *y* direction, respectively. These base vectors are aligned with the horizontal and vertical scanning directions of the SEM. Note that even though equation (8) is continuous in time, it is probed only at a set of discrete values of time (the scan times) corresponding to the scanning of pixels. This means that even though drift **D**(*t*), is smooth in time, drift distortion for each scan time **D**(*t*(***ξ***)) is by definition never smooth nor continuous (*C*^− 1^), due to the scanning process that is discontinuous in space. This discontinuity in space is observed in the pronounced change in the drift distortion from the last pixel of one row to the first pixel of the next one.

At the beginning of the first image, corresponding to *t* = 0, the drift is equal to zero. Considering that the first image holds no (imposed) mechanical deformation, it can be concluded that the motion mapping function is equal to unity (position **X**) at *t* = 0:

9 $$  {\mathbf{U}}_{1}({\mathbf{X}}) = \mathbf{0} \quad ; \quad {\mathbf{D}}(t = 0) = \mathbf{0} \quad \Rightarrow \quad {{\boldsymbol{\phi}}_{M}}_{1}(\mathbf{0}) = {\mathbf{X}}. $$

Note that, by considering equation (7), equation (4) is nonlinear in ***ξ***, i.e. an iterative solution is required for each material point **X** to find the corresponding ***ξ***. To this end, the Picard method can be utilized as a fast iterative solution method:

10 $$ {\boldsymbol{\xi}}^{P + 1} = {{\boldsymbol{\phi}}_{I}}_{i} \circ {{\boldsymbol{\phi}}_{M}}_{i} ({\mathbf{X}},{\boldsymbol{\xi}}^{P}),  $$

where *P* refers to an iteration of the Picard solution procedure with an initial guess taken from the previous iteration of the main correlation.

#### System of equations

Based on the mapping functions defined above (in equation (4)), the pixel position for each material point can be probed in each image, which results in what is often referred to as the “back-deformed image” denoted by:

**Figure:**
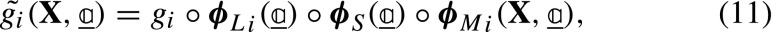

where *g*_*i*_ is image *i* and is the column of all the degrees of freedom (dof) parameterizing all the mapping functions. Note that only a part of column is associated with each of the mapping functions, but to avoid notational confusion the full column is mentioned wherever any dof is present. In absence of noise and if all the mapping functions are known, the difference between the back-deformed images is zero. However, in reality this difference, i.e. the gray scale residual, is minimized for the correct mapping functions. The gray scale residual for each image *i* is defined as: 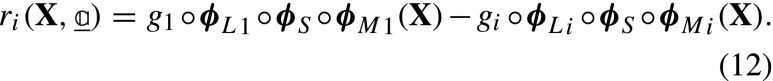


As in equation (11), all the mapping functions and consequently the residual are functions of all the dofs. For the sake of compactness, however, from here on the column of degrees of freedom is dropped in the notation of mapping functions. Minimization of the residual would lead to an ill-posed problem unless the number of unknowns is sufficiently reduced by means of regularization to the set of degrees of freedom in , as in equations (11) and (12). Note that the reference image, here chosen to be the first back-deformed image $\tilde {g_{1}}$, also incorporates artifacts, and hence it should be probed in correct positions by the corresponding artifact mapping functions.

In order to identify the unknown deformation and distortion fields, residual of all images are stacked to create a column of residual fields, and the sum of squares of this residual column $\underline {{r}}$ is minimized with respect to the degrees of freedom 
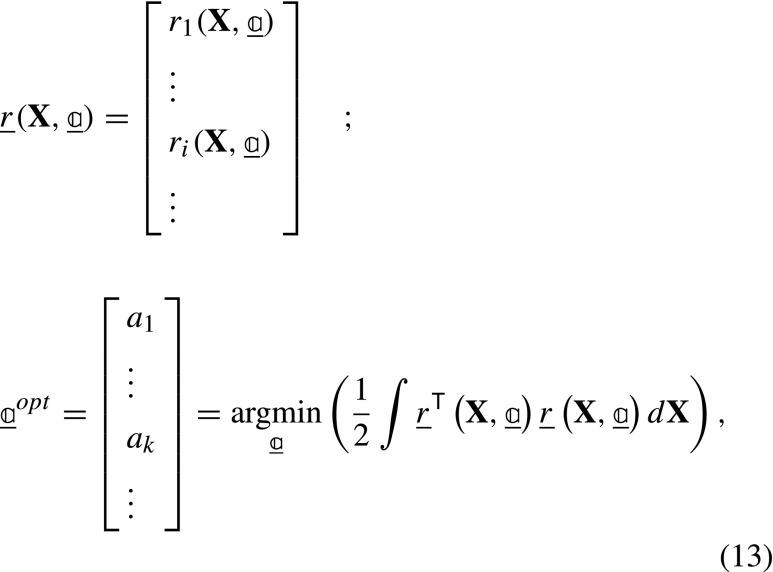


where is the set of optimal degrees of freedom minimizing the residual.

The minimization of the sum of squares of $\underline {{r}}$ implies its derivatives with respect to each degree of freedom to vanish: 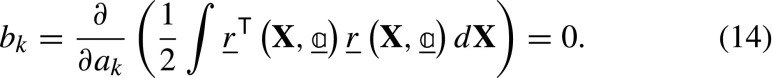


This is a set of nonlinear equations which is linearized using a Newton-Raphson solution scheme [37]: 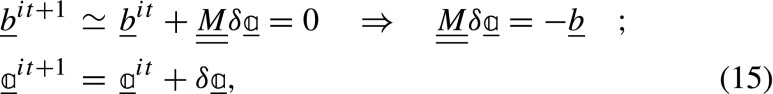
where $\underline {\underline {M}}$ is the Hessian, defined as:

16 $$  \quad {M}_{kl} = \frac{\partial {b}_{k}}{\partial {a}_{l}}. $$

The derivative of the objective function with respect to each dof, *b*_*k*_, is calculated as:

 where $\underline {{L}}$ for image *i* and dof *k* is: 
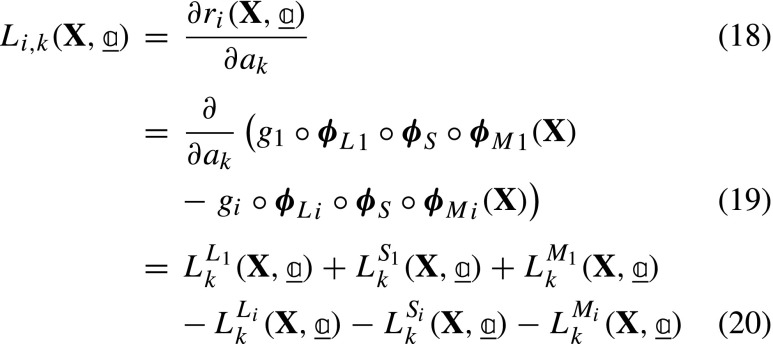
 and ${L}_{k}^{L_{i}}$, ${L}_{k}^{S_{i}}$ and ${L}_{k}^{M_{i}}$ are the derivatives of the residual for image *i* with respect to the particular dof *k* associated to ***ϕ***_*L*__*i*_, ***ϕ***_*S*_ and ***ϕ***_*M*__*i*_. Each derivative is determined by applying the chain rule:

**Figure:**
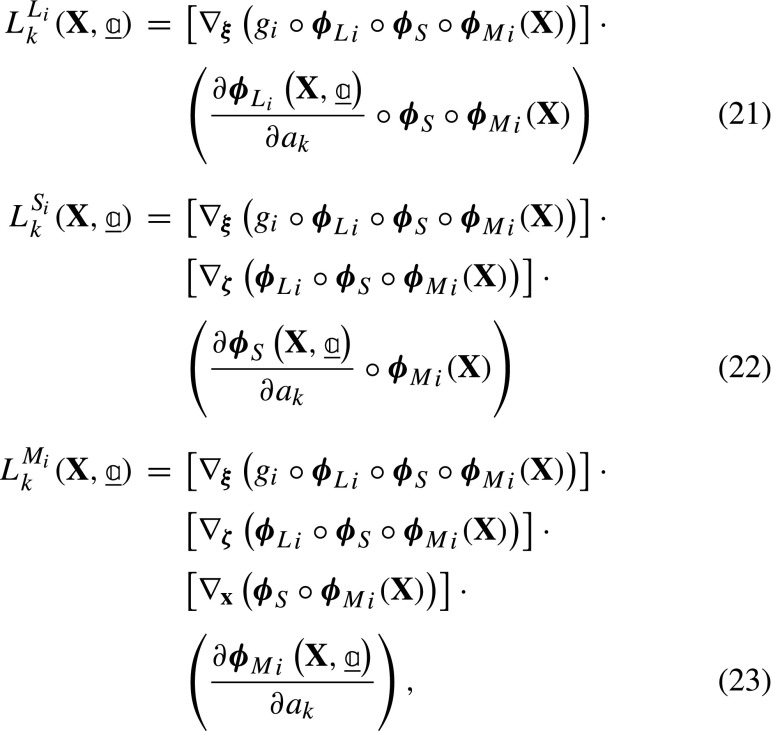

where the gradients are with respect to the corresponding subscripts indicated in each case, i.e. ***ξ*** = ***ϕ***_*L*__*i*_ ∘***ϕ***_*S*_ ∘***ϕ***_*M*__*i*_(**X**), ***ζ*** = ***ϕ***_*S*_ ∘***ϕ***_*M*__*i*_(**X**), and **x** = ***ϕ***_*M*__*i*_(**X**). For convenience of implementation, further rearrangements of equations (21) to (23) are possible, see Appendix A. In conventional GDIC, the derivatives of the displacement field with respect to degrees of freedom ($\boldsymbol {\varphi }_{k} = \frac {\partial {\mathbf {U}}}{\partial {a}_{k}}$) are referred to as basis (or sensitivity) functions. Here, in equations (21) to (23), the expressions within round brackets are the sensitivity functions, which are the derivatives of the corresponding mapping functions with respect to the degrees of freedom. The $\underline {\underline {L}}$ matrix is finally assembled as: 
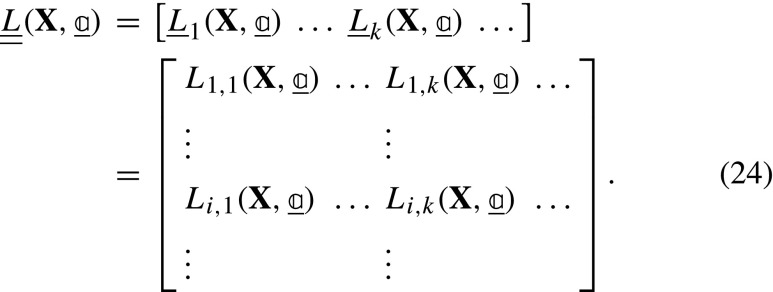
 From equations (16) and (17) the elements of the Hessian matrix, $\underline {\underline {M}}$, can be found as:

**Figure:**
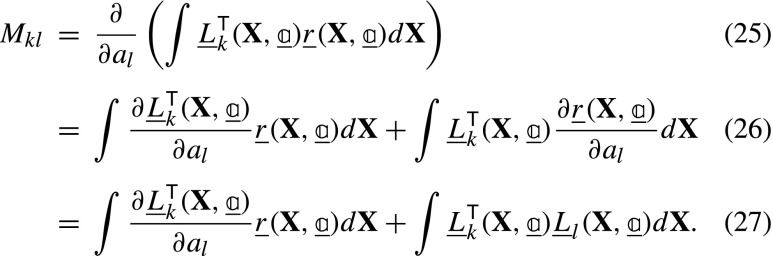

The first term in equation (27) is neglected since it contains the residual, which is small close to convergence [37], resulting in:

Note that correlation using hierarchical mapping functions takes the same amount of time as a conventional GDIC problem of the same size (in terms of number of images and dofs) if the same assumptions on the image gradient and Hessian approximation are made.

#### Reference image

Up to this point the first image, *g*_1_, was used as the reference in constructing the residual. This image may be prone to artifacts as well, and it is not more significant than any other image. Hence, a new definition of the reference image is needed, which does not introduce any bias with respect to one of the images. A weighted average of all the back-deformed images is taken to this purpose:

where *w*_*j*_ is the assigned weight to image *j* and $W = {{\sum }_{j}^{N}} w_{j}$. Using this new reference image , the residual reads:

 By choosing all the weights equal to one, a uniform average of all back-deformed images constitutes the reference image. This is important, specifically in the presence of artifacts where the first image contains distortions and needs to be back-deformed. Note that this definition does not involve an extra computational cost, since all the back-deformed images are needed to construct the residual, regardless of the definition chosen for the reference image. A more elaborate weighing scheme can be considered as well, e.g. weighing based on the inverse of the residuals. Such a scheme emphasizes images that are correlated more accurately, to construct the reference image in each iteration. The updated reference image implies only a minor change in the definition and assembly of the $\underline {\underline {L}}$ matrix: 
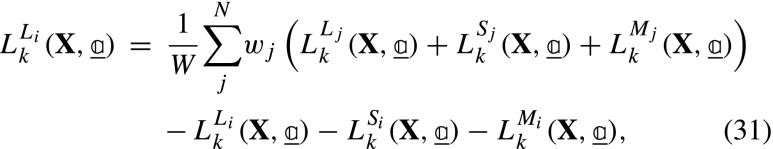


which is based on the same definitions of ${L}_{k}^{L_{i}}$, ${L}_{k}^{S_{i}}$ and ${L}_{k}^{M_{i}}$ as given in equations (21) to (23).

Note that using the average of all back-deformed images as the reference image is essential in the presence of artifacts that affect all images, including the first one. In the absence of artifacts, it does not introduce any error compared to using the first image as reference. This is demonstrated on sample 11b of the so-called “DIC challenge” [38] by considering a mesh of 20 × 10 knots (in *x* and *y* direction) of 2^*nd*^ order B-splines. Obtained results for the two definitions of the reference image differ in terms of displacements less than 3e − 4 px, which is well below the DIC accuracy.

### Regularization of the Artifact Mapping Functions

The distortion and deformation fields in the mapping functions, equations (6) and (7), are regularized by restricting this parametrization to a limited set of degrees of freedom. Depending on the expected mechanical deformation, the regularization of the mechanical deformation field may range from a low-order polynomial to a finite element-type discretization of the domain. The regularization for the distinct artifact fields, however, is determined by their nature, as discussed next.

#### Line shift artifact

In order to specify the line shift artifact field, based on the description given in the previous section and Fig. 1(b), an error function is used with four degrees of freedom for each line shift. Thus ***ϕ***_*L*_ in equation (6) can be written for one line shift as [32]:

32 $$ {\boldsymbol{\phi}}_{L} (\mathbf{x}) = \mathbf{x} + \frac{1}{2} \left( A_{x} \mathbf{e}_{x} + A_{y} \mathbf{e}_{y} \right) \left( 1 + \text{erf}\left( \frac{ (y - y_{0} )}{\frac{w}{3 \sqrt{2}}} \right) \right) , $$

where:

33 $$ \text{erf} (z ) = \frac{{2}}{\sqrt{\pi}} {{\int}_{0}^{z}} \mathrm{e}^{-t^{2}} dt, $$

and **x** = *x***e**_*x*_ + *y***e**_*y*_. The degrees of freedom are the amplitudes in x and y direction, *A*_*x*_ and *A*_*y*_, the position *y*_0_ (the row of pixels where the shift occurs) and the width of the line shift *w*. The width of the smooth line shift is included as a dof since it has been observed that such line shifts may easily span several scan lines [32].

#### Spatial distortion

In order to measure spatial distortion with minimal presumptions, a series of locally supported basis functions, such as B-splines, are chosen for regularizing the spatial distortion field. A smooth field of B-splines of order *n* discretized with *m*_*x*_ and *m*_*y*_ knots in *x* and *y* direction is considered:

34 $$ {\boldsymbol{\phi}}_{S}(\mathbf{x}) = \mathbf{x} + \sum\limits_{i = 1}^{k} \sum\limits_{j = 1}^{l} \mathbf{P}_{i,j} R_{i,j}(\mathbf{x}), $$

where *k* = *m*_*x*_ − *n* − 1, *l* = *m*_*y*_ − *n* − 1, **P** contains the components of a control point (i.e. two degrees of freedom) and

35 $$ R_{i,j}(\mathbf{x}) = \frac{B_{i,n}(x) B_{j,n}(y)}{ {\sum}_{p = 1}^{k} {\sum}_{q = 1}^{l} \left( B_{p,n}(x) B_{q,n}(y)\right)}. $$

Function *B*_*i*,*k*_(*z*) is given by:

36 $$ \begin{array}{@{}rcl@{}} B_{i,0}(z) & =& \left\{\begin{array}{ll} 1 \qquad \text{if } z_{i} \leq z < z_{i + 1} \\ 0 \qquad \text{otherwise} \end{array}\right. \\ B_{i,k}(z) \! &=& \! \frac{z \!- \!z_{i}}{z_{i+k} \!- \!z_{i}}B_{i,k-1}(z) + \frac{z_{i+k + 1} \!- \!z}{z_{i+k + 1} \!- \!z_{i + 1}}B_{i + 1,k-1}(z).\\ \end{array} $$

In the case of a point symmetric spatial distortion field, as in spherical aberrations in aberration corrected transmission electron microscopes [34, 35], globally supported basis functions, such as radial or cylindrical [28] polynomials, are chosen to describe this artifact field. The spatial distortion mapping function regularized by a radial polynomial of order *n* and a cylindrical polynomial of order *n*_*c*_ with fixed orientation *𝜃* reads:

37 $$ {\boldsymbol{\phi}}_{S}(\mathbf{x}) \!= \mathbf{x} + \sum\limits_{k = 2}^{n} {a}_{r,k} \left( \mid \mathbf{x} \mid^{k-1} \mathbf{x} \right) + \sum\limits_{k_{c}= 2}^{n_{c}} {a}_{c,k_{c}} \left( \left( \mathbf{x}\cdot\mathbf{e}'_{x} \right)^{k_{c}} \mathbf{e}^{\prime}_{x} \right), $$

where ∣**x**∣ is the Euclidean norm, and $\mathbf {e}^{\prime }_{x}$ is the rotated base vector defined as:

38 $$ \mathbf{e}^{\prime}_{x} = \cos(\theta)\mathbf{e}_{x} - \sin(\theta)\mathbf{e}_{y}. $$

The origin of the coordinate system is in the center of the image.

#### Drift distortion

Since drift distortion is defined as a rigid body motion in time projected on images through the scanning process, recall equations (7) and (8), the regularization of the drift distortion is done in time and not space. Taking into account that drift distortion is a smooth function of time, different choices can be made for its regularization, ranging from polynomials in time with globally supported sensitivity functions with few degrees of freedom, up to a B-spline discretization of the time domain with locally supported sensitivity functions and typically with more degrees of freedom. The drift distortion field regularized by an *n*^*th*^ order polynomial in time is:

39 $$ {\mathbf{D}}(t) = \sum\limits_{k = 1}^{n} \left( {a}_{2k-1} \mathbf{e}_{x} + {a}_{2k} \mathbf{e}_{y} \right) t^{k}, $$

where *t* is defined in equation (8), whereas regularization by a B-spline of order *n*, that is discretized with the knots {*t*_0_,*t*_1_,…,*t*_*m*_}∈ [0,*t*_*total*_], yields:

40 $$ {\mathbf{D}}(t) = \sum\limits_{i = 0}^{k} \left( {a}_{2i + 1} \mathbf{e}_{x} + {a}_{2i + 2} \mathbf{e}_{y} \right) B_{i,n}(t), $$

where *B*_*i*,*n*_(*t*) are given in equation (36).

### Correlation Procedure

To properly and uniquely identify the artifact fields, the following systematic procedure is proposed. Because the spatial distortion field is assumed to be time-independent and identical for all images a calibration phase is performed prior to the actual mechanical test in the so-called “spatial distortion calibration phase”. In the subsequent “mechanical test phase”, the previously measured spatial distortion field ***ϕ***_*S*_ is kept fixed, and used to directly correct the measurement of the mechanical test itself. Let us first describe the measurement of drift distortion and the scan line shift, as both artifacts need to be identified during these two phases.

Based on the similarity of the drift artifact to tension/compression/shear, for typical SEM scanning schemes, drift distortion needs to be properly distinguished from the mechanical deformations. To this purpose, following [11], two images are taken at each load step. Figure 3(a) depicts this scheme, where the horizontal axis represents the time, spanning the complete test time, and the vertical axis represents one of the components of the displacement and drift distortion. The shaded areas show the time taken for scanning each image. Considering that drift distortion is smooth and continuous in time, the only difference between the two images in each pair is due to drift distortion, while the deformation shared by the two must be due to the mechanical deformation. So, by constraining the mechanics of each image pair to be exactly identical and defining drift distortion as a smooth and continuous function in time, it is ensured that the mechanical deformation and drift distortion fields can be uniquely identified. This will be also demonstrated in the virtual experiments of “Validation by Virtual Experiments: Simple Deformation and Distortion Fields” and “Validation by Virtual Experiments: Complex Deformation and Distortion Fields”. The only constraint needed for drift is that at *t* = 0 it equals zero as mentioned before in equation (9). In this artifact corrected IDIC scheme the drift distortion in all images (including the first image) is directly measured and hence, there is no need for any extrapolation of the data, making the results more accurate. Note that both for the “spatial distortion calibration phase” and the “mechanical test phase” it is necessary to capture two images for each displacement/load step to be able to measure the drift distortion. Additionally, line shift artifacts need to be simultaneously measured, both during the spatial distortion calibration and mechanical test phase. To do so, the line shift mapping function, ***ϕ***_*L*__*i*_, is defined for each image containing any line shift artifacts (*i* = 1,2,…*n*). Since the line shift artifact, cf. equation (32), yields the same result for all positive widths smaller than one (rendering effectively the resulting system ill-posed, recall equation (32)), the corresponding dofs need to be constrained to be equal to or greater than one pixel. This is in practice achieved by means of a constrained optimization algorithm [39].

**Fig. 3:**
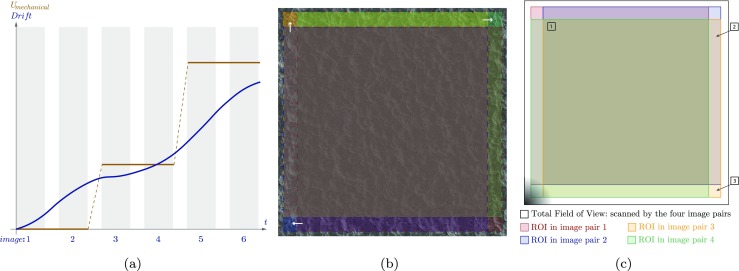
Identification of drift distortion: **a** schematic representation of the time evolution of the rigid body motion of the specimen relative to the beam, resulting in drift distortion and the load step strategy to separate the mechanical deformation from the drift artifact. Identification of spatial distortion: **b** an example of four image pairs with rigid body motions covering four corners of the field of view (FOV) for B-spline regularization of the spatial distortion. **c** Field of view of image pairs and relative positions of region of interest (ROI) in each image pair; in the center (area 1), the spatial distortion is probed by four image pairs, while close to the edges (area 2), and in the corners (area 3) it is probed, respectively, in only two and one image pairs. The shaded corner (bottom left) represents the support of a local B-spline basis function; this support should be contained in the region of interest of at least one of the image pairs to correlate its amplitude in the spatial distortion field

The spatial distortion calibration proceeds similar to Ref. [11]: a series of rigid body motions, in both *x* and *y* direction, is performed on the specimen, using the SEM stage. The consequence of a rigid body motion is that each area on the specimen experiences a different amount of spatial distortion before and after the motion. This is reflected as a field of artificial deformations which now can be measured based on the composition of the rigid body motion, described by the motion mapping function ***ϕ***_*M*_, and the spatial distortion, described by ***ϕ***_*S*_. Since the mechanical interaction is limited to rigid body motion without deformation, any measured deformation results from the spatial distortion only (when the drift distortion and possible line shifts in the calibration phase are measured as well as described above). The rigid body motions in the spatial distortion calibration phase are applied as follows. As depicted in Fig. 3(b), three steps of rigid body motion are applied consisting of a forward motion in *x*, a forward motion in *y* and finally a backward motion in *x* direction. The maximum applied rigid body motion in each direction is approximately 5% of the field of view (FOV). This is visualized in Fig. 3(c), where the FOV of the acquired image pairs is shown. Here, a part of the pattern is common to all four image pairs (regions of interest), which is positioned at different locations in individual image pairs (based on the applied rigid body motion). Each one of the colored frames in Fig. 3(c) shows where the region of interest (ROI) is located with respect to the FOV in each image pair. Note that the spatial distortion (as well as the other artifact fields) is defined in the entire FOV (not in the ROI). In order to correlate all the degrees of freedom describing the spatial distortion, all the basis functions need to have their supports in the region of interest of at least one of the considered image pairs. The shaded area in Fig. 3(c) represents an example of a locally supported basis function that satisfies this condition. To guarantee the above condition, the applied rigid body motions need to cover all four corners of the FOV, and hence the spatial distortion field. Area number 1 in this figure is probed four times, whereas areas number 2 (repeated at the four sides of the FOV) and number 3 (repeated at the four corners of the FOV) are probed only twice and once, respectively. The accuracy of the evaluation of the spatial distortion field is therefore much higher in the center (area 1). In order to maximize this area, only a limited rigid body motion should be applied (5 % of the FOV in our case). Based on this fact and since the accuracy of IDIC decreases near the edges (see e.g. Figure 6 in reference [7]), the best practice is to perform the calibration phase at about 10 % larger FOV and evaluate the spatial distortion in the central area (area 1) only. This reduced region then corresponds to the field of view of the images of the mechanical test. Note that since a change of magnification in SEM is performed by scanning over a larger or smaller field of view, as long as the beam parameters are not changed, the spatial distortion can be assumed to be constant in time. In case of globally supported basis functions for the spatial distortion (e.g. radial and/or cylindrical polynomials), it suffices to apply the rigid body motion only in the diagonal direction with a step size of almost 25% of field of view. In order to increase the accuracy of the measurement, the total diagonal rigid body motion is applied in two steps, resulting in three image pairs in total.

Note that the applied rigid body motions need to be controlled with high accuracy (of the order of 0.01 px) to accurately measure the spatial distortion in the calibration phase. Because translational control to such a high accuracy is experimentally unfeasible even with high accuracy positioning systems, rigid body motions are introduced as degrees of freedom in the motion mapping function ***ϕ***_*M*_ to measure the applied rigid body motions with high accuracy. This has consequences for the measurement of the spatial distortion field. Although zeroth order terms in spatial distortion induce a constant shift in all image pairs, this has no effect on the mechanics (measured through differences between individual image pairs). The first order terms in the spatial distortion induce a constant stretch throughout all images, which results in a zeroth order effect on the mechanics (i.e. an extra artificial rigid body motion). If the applied rigid body motions were known accurately, the first order term of the spatial distortion could be measured, but since the rigid body motion must be measured as well, the influence of the linear part of the spatial distortion is captured by the motion mapping function ***ϕ***_*M*_. Therefore, in order to prevent non-uniqueness and hence convergence issues, the spatial distortion mapping function ***ϕ***_*S*_ is constrained to be orthogonal to constant and linear functions. These constraints are applied on the spatial distortion field by means of Lagrange multipliers.

The separation of the artifact fields from the mechanical deformation field is therefore achievable based on the considerations mentioned above and summarized below:

(i) spatial distortion is a constant field in time (as long as beam parameters are not changed); it is identified during an independent calibration step in which no mechanical deformation occurs (only discrete steps of rigid body motion are applied);
(ii) drift distortion is a continuously evolving, smooth function in time, also during scanning of each image; it is distinguished from mechanical deformation which is applied in a step-wise manner between the acquisition of every two images i.e. mechanical deformation is constant in each image pair;
(iii) scan line shift artifact fields are random localized distortions with a direction dictated by the underlying scanning process; they occur discretely in time, and are distinguished from the mechanical deformation through image pairs similar to drift;
(iv) mechanical deformation is considered as an arbitrary complementary field, constant within a given loading step (i.e. constant for each image pair); i.e. no constraint is enforced on mechanical deformation.

The complete IDIC problem is solved using a Newton-Raphson scheme. The convergence of this minimization method is sensitive to the initial guess. Thus, a procedure is proposed in Appendix B to determine a set of initial guess values that guarantees convergence, starting from a zero initial guess, rendering the methodology robust. The initial guess for two sets of dofs is trivial. Considering the large values of the rigid body motions, the dofs corresponding to these motions need an initial guess that is accurate to within ± 20 px. These values are trivially known, since the rigid body motions are always manually applied in the calibration phase. Line shift amplitudes are always set to 1 px initially, since a zero value for these dofs would result in zero support for the sensitivity functions of the line shift width and position [32].

## Validation by Virtual Experiments: Simple Deformation and Distortion Fields

In order to validate the methodology introduced above, a series of virtual experiments is performed. These have the advantage over real experiments that the exact fields are known and thus the accuracy of the “measured” fields can be assessed rigorously and quantitatively. A validation based on real experimental images is presented in “Validation on Real SEM Images”. An artificially generated pattern is deformed to generate virtual images of the “spatial distortion calibration” and “mechanical test” phases. The pattern consists of three layers of randomly distributed circular Gaussian peaks. Each layer is defined as:

41 $$ F(x,y) = \displaystyle\sum\limits_{i} a e^{\left( -\frac{1}{2} \left( \frac{(x-{\mu_{x}^{i}})^{2}}{{\sigma_{x}^{2}}} + \frac{(y-{\mu_{y}^{i}})^{2}}{{\sigma_{y}^{2}}} \right) \right) }, $$

where *a* is the amplitude of each peak, *μ*_*x*_ and *μ*_*y*_ are peak center coordinates (chosen randomly), and *σ*_*x*_ and *σ*_*y*_ are standard deviations in *x* and *y* direction. The three layers have amplitudes of 0.3, 0.4 and 0.2 (in a gray scale ranging from 0 to 1), standard deviations of 35, 10 and 1.5 px, and spacings of 70, 15 and 1.5 px, cf. Fig. 7.

The calibration phase uses 6 images (3 image pairs). The second and third image pairs have been obtained by applying diagonal rigid body motions of 70 and 140 pixels (corresponding to 27*%* of the field of view), respectively. Similarly, the mechanical test phase uses 3 image pairs. The first image pair is free of any mechanical deformation, serving as the mechanics reference. The last pair incorporates the mechanical displacement field, the vector amplitude of which is depicted in Fig. 4(a), and the second pair bears exactly half of that displacement field. The applied deformation corresponds to a piecewise constant strain field, with deformation gradients in the four areas given in Fig. 4(a). The deformed shape is shown as well. Even though this deformation is rather simple, it looks similar to a typical drift distortion (tension in *y* direction and shear), thus constituting a challenging case for distinguishing the drift artifact from the mechanics.

**Fig. 4:**
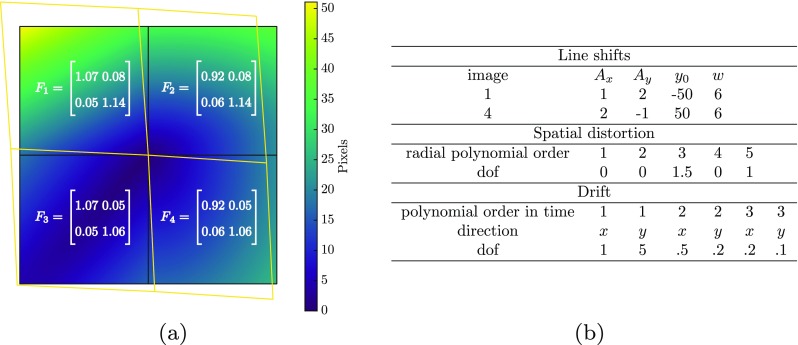
Virtual experiment input. **a** Applied virtual mechanical field: amplitude of displacement fields and corresponding deformation field in image 5 and 6, i.e. the last image pair in the series of images for the mechanical test phase. The four quarters indicate areas of constant deformation gradient. **b** Degrees of freedom for the input virtual artifact fields

All virtual images in the calibration and mechanical test case are also distorted by typical artifact fields. The spatial distortion is equal in both the calibration and mechanical deformation phase. The drift distortion and line shift artifact fields are identified independently in both phases without mutual influence. Hence, the same artifact fields are implemented in both phases for the sake of simplicity. The virtual artifact fields are applied through the hierarchical mapping functions, replicating the imaging process in an SEM. A third order polynomial in time describes the input virtual drift distortion field. The spatial distortion field, which is applied equally to all the images, is a radial polynomial of order 5. Two scan line shifts are applied, respectively, to image 1 and 4. Note that the existence of a line shift in the first image makes it even more challenging to accurately measure drift distortion in this image. Each line shift is described by a smooth error function with amplitudes up to ± 2 pixels (px) in *x* and *y* direction and a width of six pixels. The degrees of freedom corresponding to each of the artifact fields are listed in table of Fig. 4(b). Finally, Gaussian noise with a standard deviation of 0.1*%*, 1*%*, 2*%*, 5*%* and 10*%* is added to all the images, yielding five image series (each with 12 images) with the same deformation and distortion fields, but different noise levels.

In the next subsections, first a correlation case is discussed where the exact same regularization functions as the input distortion and deformation fields are chosen, followed by three studies on the effect of noise, regularization of spatial distortion field and regularization of drift distortion in time.

### Correlations with Nearly Optimal Regularizations

The identification of the fields (calibration and mechanical phase) is done on the virtual images with 1*%* noise using the same regularization of mapping functions that were used to generate the virtual images. This is the optimal choice of regularization since there are the exact number of dofs needed to identify the virtual distortion and deformation fields in the virtual images. To incorporate the rigid body motion in the spatial distortion calibration phase, the mechanical displacement field, **U**, in the motion mapping function ***ϕ***_*M*_ is chosen to be constant. For the mechanical test phase, this field is regularized by 4 × 4 elements of first order B-splines. In both the calibration and test phase, the correlations are initiated with a straightforward initial guess and performed following the steps discussed in Appendix B.

The correlation of the spatial distortion calibration phase is performed with a (trivial) zero initial guess. The convergence is robust and monotonic. In the calibration phase, the spatial distortion field is measured alongside the drift distortion and the line shift artifacts to guarantee the accuracy of the measurement. Figure 5(a), (b) and (c) depict the amplitude of the measurement error of the artifact fields in the calibration phase. Notations used for the mean absolute value of the vector amplitude of the errors in the measured displacement and artifact fields are: $\bar {\mathcal {E}}_{U}$, $\bar {\mathcal {E}}_{D}$, $\bar {\mathcal {E}}_{S}$, $\bar {\mathcal {E}}_{L}$ for mechanical displacement (in this phase applied rigid body motion), drift artifact, spatial distortion and line shift artifact fields, respectively. Figure 5(a) shows the error for the identified drift distortion in the first image. The mean error ($\bar {\mathcal {E}}_{D} = 0.0005$ px) and the maximum value (0.0009 px) are both well below the accuracy of DIC, which is in the order of 0.01 px, depending on the continuity of the regularization and the spatial resolution [40]. These very low errors in the first image are explained by the fact that the drift distortion is constrained to zero for the first pixel (top right) of the first image, yielding zero error in this pixel. Note that the measurement of drift distortion in the first image with this accuracy is only possible with the adopted time regularization for drift distortion, equation (7), and the coupled framework employed in the current study. The error of the spatial distortion is shown in Fig. 5(b) with mean absolute and maximum values of 0.0045 and 0.008 px, respectively. Figure 5(c) shows the error of the line shift artifact of image 4. The mean error for this field is $\bar {\mathcal {E}}_{L} = 0.0015$ px. Note that the maximum value of the error in the line shift artifact field is restricted to the width of the line shift. This is due to the slightly lower sensitivity to dofs related to the position and width of line shifts, since the supports of the sensitivity functions related to these dofs are as small as the width of the line shift (a few pixels). The error fields in Fig. 5 need to be compared with their corresponding artifact fields. The maximum value of the input artifact fields corresponding to drift distortion in image 1, spatial distortion, and line shift in image 4 are 0.68, 9.9 and 2.23 px, respectively, while the inaccuracy with which these fields have been identified is roughly three orders of magnitude lower. This emphasizes the difference in the scale of the artifacts and the error in evaluating them.

**Fig. 5:**
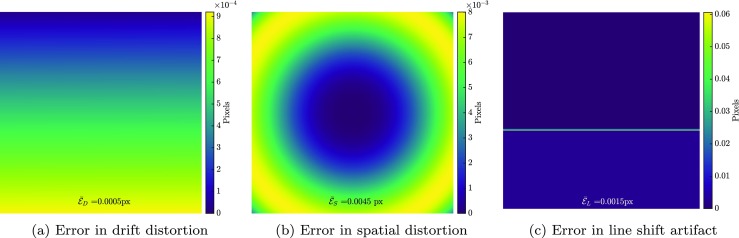
Error fields in the calibration phase: **a** error amplitude of drift measurement in image 1, **b** error amplitude of spatial distortion measurement **c** error amplitude of line shift artifact measurement in image 4

The spatial distortion field measured in the calibration phase (with mean error of $\bar {\mathcal {E}}_{S} = 0.004$ px) is subsequently used to correlate the images of the mechanical test phase. In this correlation the spatial distortion mapping function is activated, so that the images are directly “corrected” for the spatial distortion effect (with fixed dofs) while the drift distortion and line shift artifacts are measured alongside the mechanics. This step is again initiated with a zero initial guess, entailing robust and monotonic convergence. Figure 6(a) illustrates the residual field of image 6 (normalized with respect to the dynamic range of the image) in the correlation while Fig. 6(b) shows the same residual if all artifacts are neglected (using conventional GDIC). The mechanical displacement field is measured with high accuracy, see Fig. 6(c), which shows the amplitude of the error of the mechanical displacement field. The mean absolute value of this error field is $\bar {\mathcal {E}}_{U} = 0.005$ px. To assess the accuracy of the results, Fig. 6(d) shows the amplitude of the error in the mechanical displacement field, if all artifacts are neglected (using conventional GDIC with the same regularization for the mechanical deformation field). Note that both the mean absolute value of the error and the range of the color bar are more than two orders of magnitude smaller when the artifact corrections are included. Figure 6(e) and (f) show the amplitude of error in the drift distortion in the first image (mean error of $\bar {\mathcal {E}}_{D} = 0.0006$ px) and the line shift artifact in the fourth image (mean error of $\bar {\mathcal {E}}_{L} = 0.001$ px), respectively. Note again the difference in the mean absolute value and the color bar range of these fields and the case of Fig. 6(d).

**Fig. 6:**
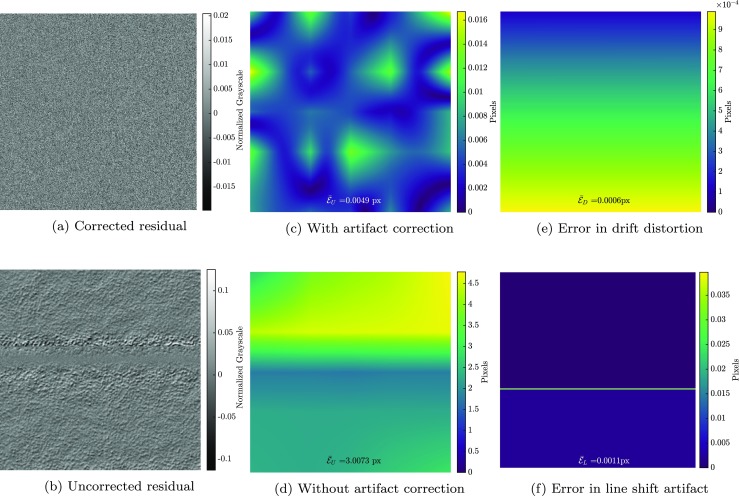
Mechanical test phase results: residual field in image 6 (normalized with respect to the dynamic range of the image) **a** with and **b** without artifact correction. Error amplitude of the mechanical displacement field in image 6 **c** with and, **d** without artifact correction, **e** error amplitude of drift distortion in image 1, and **f** error amplitude of line shift artifact in image 4

### Noise Robustness

The same procedure and parameters as described in “Correlations with Nearly Optimal Regularizations” are used to analyze the image series with different noise levels. For each noise level, first the calibration images are correlated to identify the spatial distortion (with an error that increases with increasing noise level), which is then used to correlate the images of the mechanical test phase. Figure 7 shows the mean absolute value of the error in the mechanical displacement fields of the main test, as a function of the noise level. The proposed methodology remains robust in the presence of noise, which is vital in analyzing SEM images, where the noise levels are typically (much) higher than in optical images. A noise level of 2*%* results in a mean error in the mechanical displacement equal to $\bar {\mathcal {E}}_{U} = 0.007$ px, which is remarkably good considering the typical DIC accuracy limits.

**Fig. 7:**
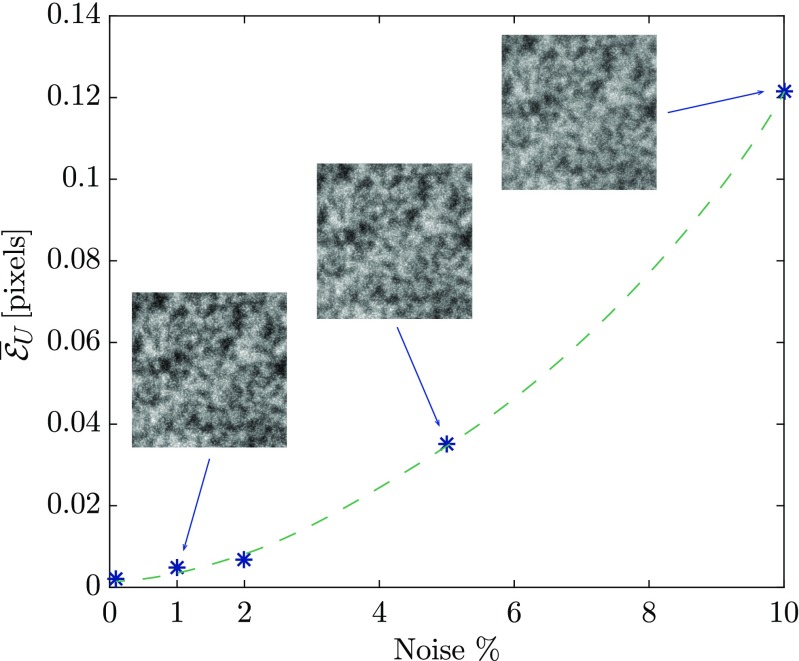
Mean absolute value of the amplitude of error in the measured mechanical displacement field as a function of the noise in the images of both the calibration and main test phase, where the noise is defined by its standard deviation as a percentage of the dynamic range of images. The images refer to the virtual patterns for 1*%*, 5*%* and 10*%* of noise

### Spatial Distortion Regularization Study

Using the images with 1*%* noise, different regularizations of the mapping function for spatial distortion are used to repeat the correlation in the calibration phase. This demonstrates that more general regularization choices for the spatial distortion still lead to high accuracy. The drift distortion and line shift artifact fields are regularized in the same way as in the previous section. Figure 8(a) depicts the different regularization cases and the mean absolute value of the error amplitude of the measured spatial distortion field for each. In the first three cases, spatial distortion is regularized by radial polynomials. In the last four cases a series of cylindrical functions in both *x* and *y* direction are added, e.g. case five includes radial polynomial of order 9, as well as cylindrical functions in *x* direction of order 3 and cylindrical functions in *y* direction of order 3, i.e. eight dofs in total. First order terms are not included in the radial nor cylindrical polynomials because of the discontinuity of the gradient fields at the origin, which would trigger convergence problems. Note that both the *x* and *y* components of each term of the radial polynomials are anti-symmetric with respect to the *y* and *x* axis, respectively. This is opposite to the normal polynomials, where the even order terms are symmetric and the odd order terms are anti-symmetric. Based on this fact, radial monomials of consecutive order have quite similar shapes, which if included all, make the system ill-conditioned or even ill-posed. Accordingly, only odd number order terms of radial polynomials are included in the regularization of the spatial distortion. It is observed, in Fig. 8a, that the error in measurement of the spatial distortion field remains in an acceptable range (less than $\bar {\mathcal {E}}_{S} = 0.018$ px) in the presence of extra radial functions in the regularization of the spatial distortion mapping function. Moreover, adding cylindrical functions to the regularization has a negligible effect.

**Fig. 8:**
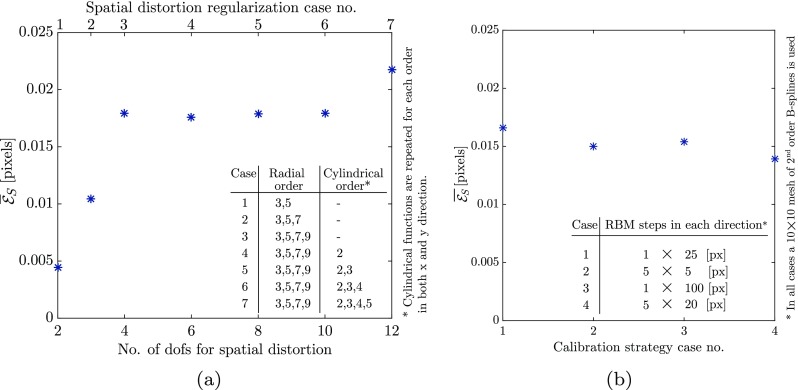
Spatial distortion studies: **a** Mean absolute value of the amplitude of the error vector in the measured spatial distortion field as a function of number of degrees of freedom used to regularize the spatial distortion field in the calibration phase. The regularization functions used in each case are described in the legend. **b** Mean absolute value of the amplitude of the error vector in the measured spatial distortion field as a function of strategy used for calibration phase in terms of rigid body motion (RBM) steps conducted. All four cases are correlated with a B-spline regularization of spatial distortion field

Four additional virtual experiments are performed to study the influence of the number and the magnitude of the rigid body motion steps in the calibration phase, on the evaluation of spatial distortion. In all the cases the images contain only rigid body motions and radial spatial distortions, and the correlations are done using a 10 × 10 mesh of second order B-splines for spatial distortion and zeroth order polynomials for mechanics. All cases follow the strategy of Fig. 3(b) with rigid body motions given by: (i) one 25 px step in each direction, (ii) five 5 px steps in each direction, (iii) one 100 px step in each direction, and (iv) five 20 px steps in each direction. The error in the evaluation of the spatial distortion in all four cases is equal and in line with the accuracy expected for DIC, see Fig. 8(b).

### Drift Distortion Regularization Study

The images with 1*%* noise are used to perform a series of correlations (of the mechanical test phase) changing the regularization of drift distortion in time, while using the regularization functions for mechanics, line shifts, and spatial distortion field used in “Correlations with Nearly Optimal Regularizations”. This analysis reveals the accuracy of the mechanical deformation measurements despite the more general (more dofs) regularization of drift artifact. Three cases of polynomials in time (third, fourth and fifth order) and four cases of second order B-spline functions in time are used for drift distortion regularization functions, all reported in Fig. 9. The B-spline cases consist of different cases of discretization of time with five to nine knots, evenly distributed over time.

**Fig. 9:**
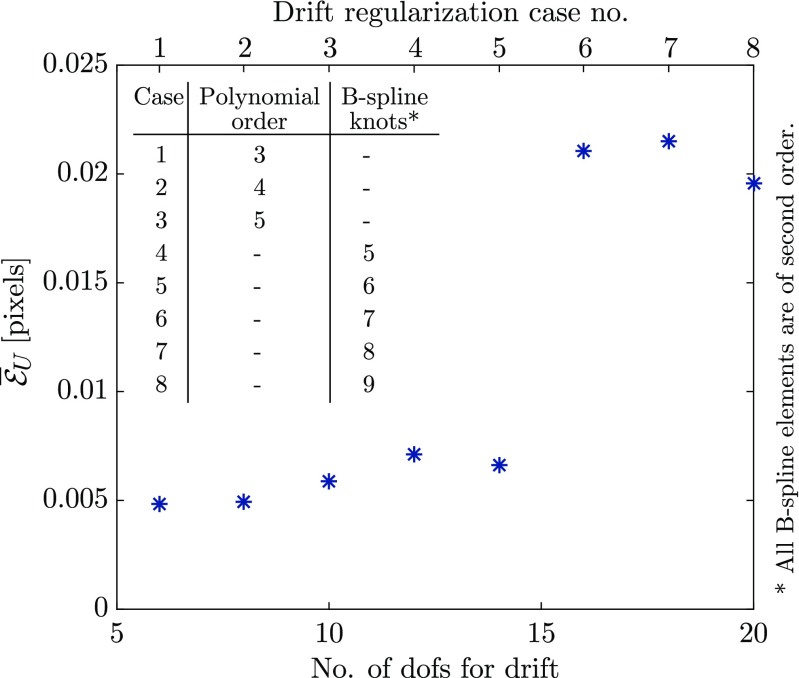
Mean absolute value of the amplitude of the error vector in the measured mechanical displacement field as a function of number of degrees of freedom used to regularize the drift artifact field in the mechanical test phase. The regularization functions used in each case are described in the legend

The cases of drift distortion regularized with up to 14 dofs (second order B-splines with six knots) result in less than 0.007 px of absolute mean value of error in the measured mechanical displacement field. The error for the higher-order regularization cases is higher but remains around 0.02 px.

### Combination of Higher Order Regularizations

A final case is examined, combining the effects of noise and higher order regularization of spatial distortion and drift distortion. Images with 2*%* noise are used for both calibration and mechanical test phases. The spatial distortion is regularized with the Case 3 of “Spatial Distortion Regularization Study” (radial polynomials of order 3, 5, 7 and 9). The spatial distortion found is used for the main mechanical test correlation in which drift distortion is regularized with Case 5 of “Drift Distortion Regularization Study” (second order B-splines in time with six knots). A mean absolute value of the amplitude of error of the mechanical displacement field of 0.019 px results, which is still a good value when compared to the typical accuracy associated with DIC.

## Validation by Virtual Experiments: Complex Deformation and Distortion Fields

The second set of virtual experiments considers a more realistic spatial distortion field, mechanical deformation field, and drift distortion that are all correlated with a generic B-spline regularization, a scan line shift in each image, and more realistic patterns for SEM-DIC.

Figure 10(a) shows a strain field measured using SEM-DIC by Stinville et al. [31], exhibiting high strain gradients and localizations. The figure reports *ε*_*xx*_ measured in a field of view 85*μm* of a René 88DT (a commercial polycrystalline nickel-based super-alloy) under 0.98% of global strain, which is obtained by stitching several measurements of separate scans. Local DIC with 21 pixel (0.4*μm*) subset size, step size of 3 and strain window of 15 pixels was used to make this measurement. The slip band pattern observed in the diagram has inspired the complex mechanical deformation field for the virtual experiments of this section. It features parallel localization bands with an orientation of 45^∘^ that span the entire width of the image, which is a challenging case for the accurate measurement of scan line shifts [32]. Figure 10(b), (c) and (d) depict a zoomed area of Fig. 10(a) inside which the individual strain components *ε*_*xx*_, *ε*_*yy*_ and *ε*_*xy*_ are shown, corresponding to a background stretch of 0.5*%* in *x* and compression of − 0.25*%* in *y* direction in addition to the 45^∘^ shear bands. The strain amplitudes, the width (60 px) and the spacing (200 px) of the shear bands are closely matching those of Fig. 10(a). Note that the size of zoomed area as in Figs. 10(b), (c) and (d) is comparable to each scan/DIC measurement used by Stinville et al. [31].

**Fig. 10:**
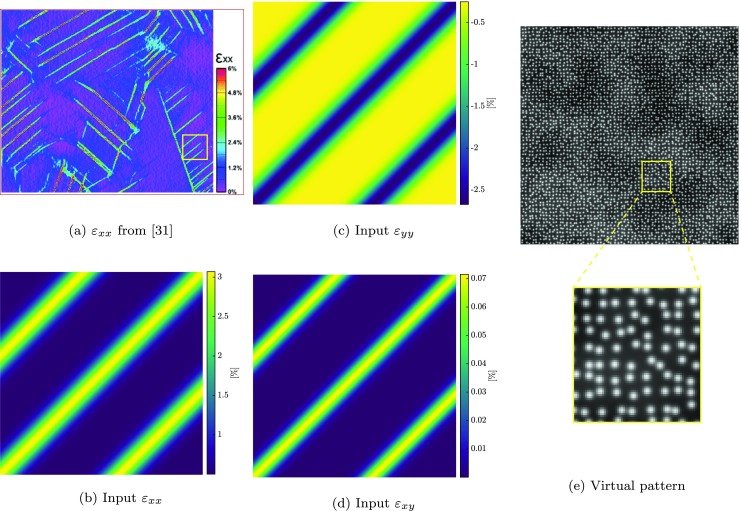
Reference mechanical deformation, taken from [31], used in the “complex” virtual experiments. **a** An example of a DIC measurement on SEM images, taken from [31], exhibiting high strain gradients and localization bands. The yellow frame depicts the zoomed area used as the reference mechanical deformation in the “complex” virtual experiments. **b**, **c** and **d** The *ε*_*xx*_, *ε*_*yy*_ and *ε*_*xy*_ fields used for the mechanical deformation in the virtual experiments of this section, exhibiting localization bands spanning the whole image with an orientation of 45^*o*^, in addition to a background stretch of 0.5*%* in *x* and compression of − 0.25*%* in *y* direction. **e** Virtually generated pattern used in “Validation by Virtual Experiments: Complex Deformation and Distortion Fields”, and the zoomed view

Figure 11(a) and (b) show spatial distortion fields that were experimentally measured, from images at 200 × magnification, in the work of Sutton et al. [11]. Figure 11(c) and (d) show the spatial distortion fields used for the virtual experiments of this section, closely matching those of Fig. 11(a) and (b).

**Fig. 11:**
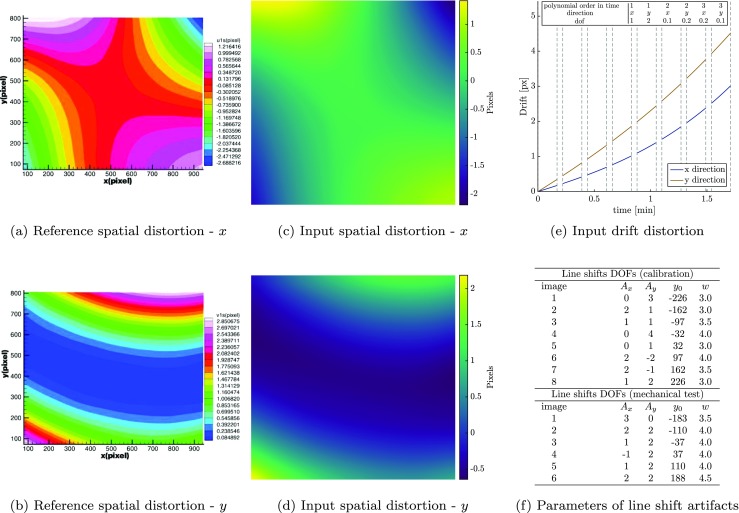
Input spatial distortion used in the “complex” virtual experiments: **a** and **b** the SEM spatial distortion fields in *x* and *y* direction experimentally measured by Sutton et al. [11]; **c** and **d** the spatial distortion fields used for the complex virtual experiments in *x* and *y* direction, respectively, matching the fields of (**a**) and (**b**). **e** Evolution of drift distortion in time and, **f** list of parameters characterizing the line shift artifacts, used for the generation of all the virtual images in the calibration (four image pairs) and mechanical test phases (three image pairs)

A third order polynomial in time (similar to Sutton et al. [11]) is used to describe the drift distortion, cf. Fig. 11(e), and one scan line shift is present in each image. The line shifts are equally spaced and distributed among the images. Their amplitudes in both *x* and *y* direction are randomly chosen from a normal distribution with a mean of 1.5 px and a standard deviation of 1.75 px. The widths are taken from a normal distribution with a mean of 7 px and a standard deviation of 1.5 px. The parameters used for generating the scan line shifts are listed in Fig. 11(f).

The images for the spatial distortion calibration phase are generated as described in “Correlation Procedure” with rigid body translations of 25 px (corresponding to more than 4.2% of the field of view). The subsequent mechanical test phase consists of three image pairs. The last image pair carries the full mechanical deformation (as depicted in Fig. 11), the second image pair contains half of this deformation, whereas the first pair is undeformed.

In order to measure complex mechanical fields with high strain gradients, a fine discretization of the 2^*nd*^ order B-spline mesh is required. This makes the problem more sensitive to the virtual pattern used in the images, which is generated as follows. The first two layers consist of randomly distributed circular Gaussian peaks with amplitudes of 0.2 and 0.1, standard deviation of 35 and 10 px, and spacings of 70 and 20 px, respectively. The last layer is generated by a randomly perturbed regular grid of isotropic Gaussian peaks of 0.7 amplitude and a standard deviation of 1.5 px. The considered grid has a spacing of 8 px, whereas the position of each speckle is perturbed by a random value between -2 and 2 px, cf. Fig. 10(e). The higher contrast and the more unified distribution of the finest speckles makes this pattern more suitable for the evaluation of the proposed method with complex deformation and distortion fields. Moreover, this pattern is more realistic for SEM-DIC, where micro or nano particles are used [2], while there are always some long-range brightness variations due to e.g. different crystals in a poly-crystalline material. Images of 583 × 583 and 513 × 513 px with 1% noise level are generated for the calibration and mechanical test phases, respectively — recall Fig. 3(c) and the discussion therein.

The spatial distortion field is regularized by a 10 × 10 mesh of second order B-splines. The edge and corner elements are chosen to be twice as large as the remaining ones to reduce the higher sensitivity to the edges. A 30 × 30 mesh of second order B-splines is used to parametrize the mechanical displacement field, where a ratio of 1.5 is used to scale the edge elements. Drift is regularized in time by 6 knots of second order B-splines (case 5 in Fig. 9), in both the calibration as well as the mechanical test phase. Scan line shifts are all identified and assigned to the corresponding images in a pre-correlation step for both the spatial distortion calibration and the mechanical test, see Appendix B. The error function of equation (32) is used to define the line shift mapping function for each image. In both the calibration and test phase, the correlations are initiated with straightforward initial guesses and performed following the steps discussed in Appendix B.

Figures 12(a) and (b) show the error in the determination of the spatial distortion field in the calibration phase, in *x* and *y* direction. The mean absolute value of these errors over the central area of the field of view (area 1 in Fig. 3(c), which is the measured area for the mechanical test phase) are $\bar {\mathcal {E}}_{S_{x}} = 0.012$ px and $\bar {\mathcal {E}}_{S_{y}} = 0.024$ px for the *x* and *y* direction respectively.

**Fig. 12:**
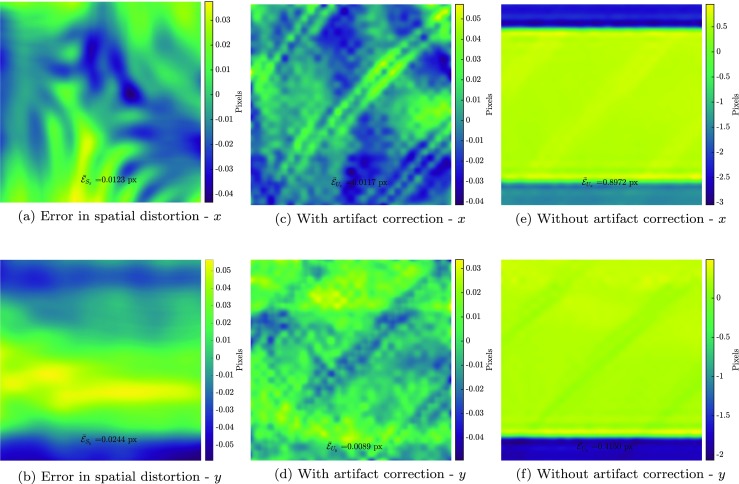
Error in virtual experiments with complex deformation and distortion fields (input fields taken from [31] and [11]). **a** and **b** spatial distortion, **c** and **d** mechanical displacement with artifact correction, **e** and **f**, the error in mechanical displacement without artifact correction (using conventional GDIC with the same discretization for the mechanical deformation field)

The error in the resulting mechanical displacement field, obtained from the mechanical test phase, is reported in Fig. 12(c) and (d). The mean values of these error fields are $\bar {\mathcal {E}}_{U_{x}} = 0.012$ px and $\bar {\mathcal {E}}_{U_{y}} = 0.009$ px, which is approximately equal to the general DIC accuracy, indicating that all artifacts have been captured with high accuracy. For comparison, the errors in the mechanical displacement field for the case where the artifacts are ignored are shown in Fig. 12(e) and (f). The mean absolute values are, $\bar {\mathcal {E}}_{U_{x}} = 0.897$ px and $\bar {\mathcal {E}}_{U_{y}} = 0.415$ px, i.e. a factor of 75 and 46 higher, thereby underlining the importance of proper artifact correction.

Figures 13(a), (b) and (c) depict the error in the measurement of the *ε*_*xx*_, *ε*_*yy*_ and *ε*_*xy*_ strain components by the proposed artifact corrected framework. The mean values of these errors are $\bar {\mathcal {E}}_{\varepsilon _{xx}} = 0.06 \%$, $\bar {\mathcal {E}}_{\varepsilon _{yy}} = 0.05 \%$ and $\bar {\mathcal {E}}_{\varepsilon _{xy}} = 0.03 \%$, respectively. Figure 13(d), (e) and (f) depict the same strain components for the case with ignored artifacts, with mean values of $\bar {\mathcal {E}}_{\varepsilon _{xx}} = 0.16 \%$, $\bar {\mathcal {E}}_{\varepsilon _{yy}} = 0.85 \%$ and $\bar {\mathcal {E}}_{\varepsilon _{xy}} = 0.87 \%$. The considered line shift artifacts typically affect the *ε*_*yy*_ and *ε*_*xy*_ components and have a negligible effect on *ε*_*xx*_. Note the large localized errors in *ε*_*yy*_ and *ε*_*xy*_ if artifacts are not corrected for, while no trace of such large errors are found in the corrected case.

**Fig. 13:**
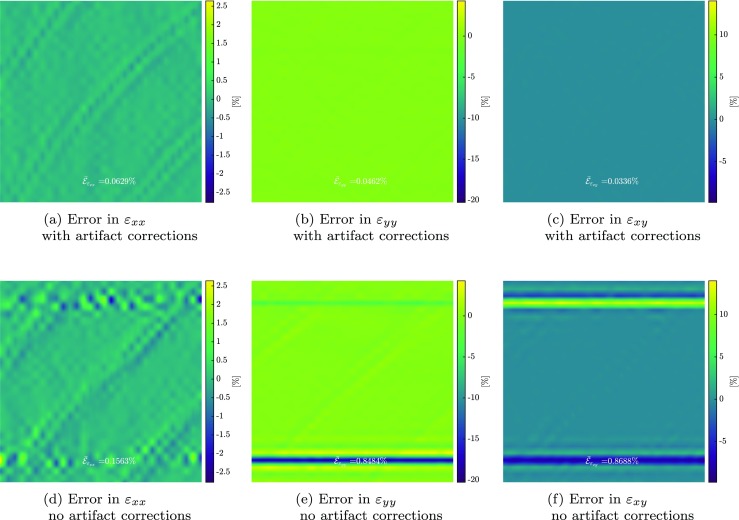
Error in strain components in virtual experiments with complex deformation and distortion fields (input fields taken from [31] and [11]). Error in **a***ε*_*xx*_, **b***ε*_*yy*_ and **f***ε*_*xy*_ with artifact correction. Error in **a***ε*_*xx*_, **e***ε*_*yy*_ and **f***ε*_*xy*_ without artifact correction (using conventional GDIC with the same discretization for the mechanical deformation field)

## Validation by Virtual Experiments: Application to Real SEM Patterns

In this section the proposed methodology is validated using virtual experiments in combination with speckle patterns from real SEM images. To this end, the virtual experiments of the previous section (with complex spatial distortion and mechanics obtained from experiments of [31] and [11], respectively) is repeated using the patterns from Fig. 14. The same virtual deformation and distortion fields are used to virtually deform the SEM patterns mentioned above, and the mechanical deformation field as well as the artifact fields are measured in the same way. Figure 14(a) provides a regular SEM-DIC pattern, which is obtained by sputter coating of a silicon substrate by an Indium-Tin target and then heat treated to the melting point of the alloy (98 ^∘^C) to create a pattern consisting of spheres [41]. Note that this is only a suitable pattern if used at a high magnification. For the experimental validation in “Validation on Real SEM Images”, we also need speckle patterns that are suited for simultaneous analysis at multiple scales. Therefore, a multiscale pattern, based on the fractal growth of copper during electro-deposition, is used as well. Figure 14(b), (c) and (d) depict this multiscale pattern imaged at three different magnifications corresponding to 10, 50 and 600 *μm* horizontal fields of view (HFV). The result is an acceptable, though not optimal, DIC pattern at multiple scales.

**Fig. 14:**
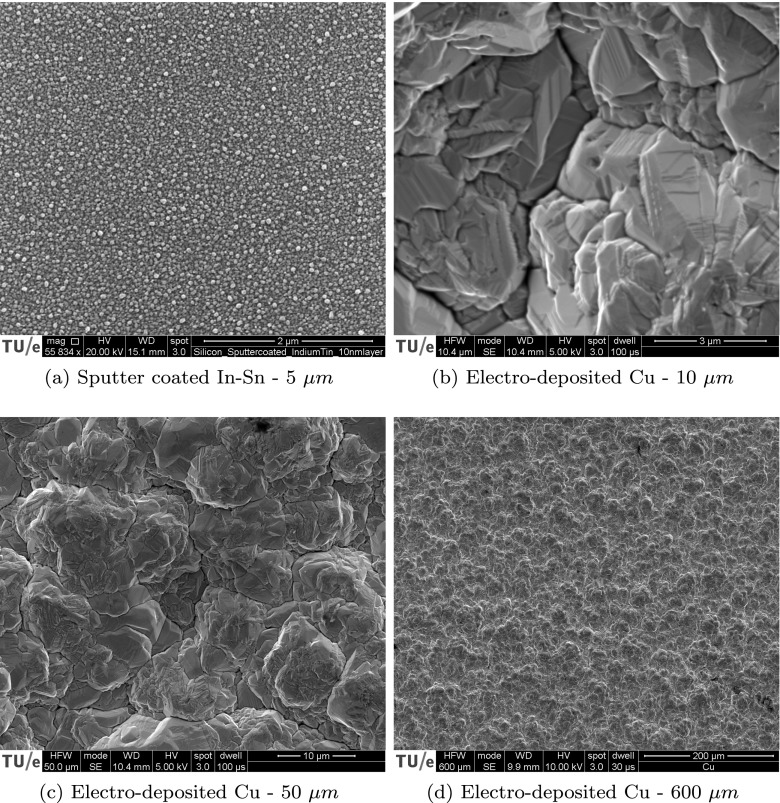
Four realistic SEM patterns used in virtual experiments with complex deformation and distortion fields. **a** regular small scale SEM-DIC pattern obtained by Indium Tin sputter coating on a silicon substrate imaged in HFV of 5 *μm*. Electro-deposited copper imaged with an FEI Quanta 600 SEM, in secondary electron contrast mode, visualized in three different magnifications corresponding to **b** 10, **c** 50, and **d** 600 *μm* horizontal field of view (HFV). The fractal growth, resulting from the electrodeposition of copper, provides a natural DIC pattern at different scales

All images are taken in a FEI Quanta 600 SEM, in secondary electron contrast mode, with 1024 × 884 pixels. The Indium-Tin sputter coated image is taken at 15*mm* working distance, with 20*kV* beam voltage and 100*μs* dwell time. The electro-deposited copper images are taken with 10 *mm* working distance while the ones with 10 and 50 *μm* horizontal field of view are acquired with 5*kV* beam voltage and 100*μs* dwell time, and the images with 600*μm* HFV with 10*kV* and 30*μs*.

The obtained results for the spatial distortion calibration phase and the mechanical test phase are summarized in Fig. 15 for the four real SEM patterns of Fig. 14 as well as for the virtual pattern of Fig. 10(c). In particular, Fig. 15(a) shows the errors in the spatial distortion field $\bar {\mathcal {E}}_{S}$ (corresponding to the spatial distortion calibration phase), whereas Figs. 15(b) and (c) show the error in the mechanical displacement $\bar {\mathcal {E}}_{U}$ and strain field $\bar {\mathcal {E}}_{\varepsilon }$ (corresponding to the mechanical test phase). In all the three graphs, the vertical axis represents the mean absolute error values, whereas error bars reflect their maxima and minima. Clearly, the regular SEM-DIC pattern (in Fig. 14(a)) achieves the same high accuracy as that of the virtual pattern of Fig. 10(e), showing that the method is not very sensitive to the precise pattern. Indeed, even the suboptimal multiscale pattern (of Figs. 14(d) – (b)) reveals only a somewhat lower accuracy and higher scatter. Still, the obtained accuracy is adequate for practical purposes. The reduced accuracy with the electro-deposited copper pattern is explained by the fact that these patterns show low local spatial contrast in certain areas. Such local lack of contrast leads to higher error in corresponding elements in the B-spline regularization of both spatial distortion and mechanical deformation fields. Due to the finer discretization of the mechanical field these local errors are observed more in the mechanical deformation error, explaining the larger maximum and minimum values. This comparison suggests that higher errors are to be expected for the patterns based on the electro-deposited copper film or patterns with poor contrast in general. On the other hand, such a suboptimal pattern provides the possibility to measure at different scales. Note that the error in spatial distortion is higher at lower magnification, where the spatial distortion is larger.

**Fig. 15:**
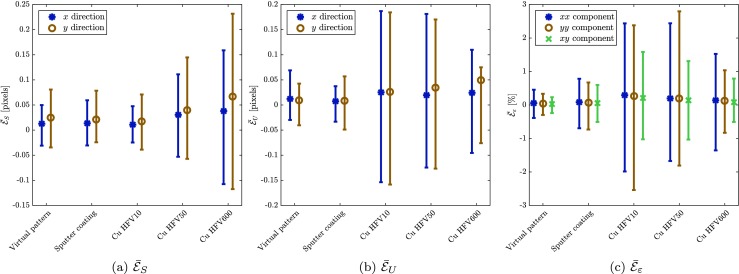
Influence of the SEM patterns (Fig. 14) on the error in **a** spatial distortion, **b** displacement and **c** mechanical strain measurements corresponding to virtual experiments with complex deformation and distortion fields taken from [31] and [11]. The horizontal axis refers to the patterns of Figs. 14(a)–(d) and 10(e) respectively. The vertical axis represents the mean absolute value of the error, whereas the error bars reflect the minimum and maximum values

## Validation on Real SEM Images

Finally, the proposed methodology is used to assess the accuracy with which the artifacts can be measured in a series of real SEM images. In a real mechanical test, it is unfeasible to apply a higher-order mechanical deformation field which is known a priori with sufficiently high accuracy in order to validate the measurement accuracy. Therefore, instead, the experimental validation is performed by evaluating in detail the measured distortion (scan line shifts, spatial distortion, drift distortion) fields, as well as the improvement in the image residuals obtained by applying the artifact corrections. To this end, a simple rigid body motion, in which the mechanical deformation is known to be zero everywhere, is applied to the specimen, as done in the spatial distortion calibration phase. The calibration phase is performed, as described in Fig. 3(b). Three magnifications are considered, corresponding to 10, 50 and 600 *μm* HFV as depicted in Figs. 14(b), (c) and (d). Two series of images are taken at each magnification, to assess the reproducibility.

In all correlations performed, the spatial distortion field is regularized by a 10 × 10 mesh of second order B-splines. Drift distortion is regularized by 10 knots of second order B-splines in time. An error function is assigned to each detected line shift (see Appendix B), and zeroth order polynomials are used for the mechanics to capture the applied rigid body motion between each image pair.

Figs. 16(a)–(b) depict the measured spatial distortion fields determined from the two measurements at the lowest magnification (600*μm* HFV). The two measurements (taken on the same day) match well. This reproducibility supports the assumption that the spatial distortion is time-independent, as long as the electron beam parameters are not changed. The difference of these two measurements is shown in Fig. 16(e) and (f). The mean absolute value of these error fields amounts to 0.015 px in *x* and, 0.010 px in *y* direction, showing high reproducibility of the spatial distortion measurements. Such low reproducibility error values alongside the low residual fields of the correlations (discussed below) indicate the high accuracy of the measurements. For more quantitative analysis, the diagonal of the spatial distortion fields in the *x* direction measured in all six tests at three magnifications are plotted on the same physical scale in Fig. 17. Note that because the mean of the spatial distortion fields is by definition zero, as explained in “Regularization of the Artifact Mapping Functions”, all curves in Fig. 17 are vertically shifted to zero in the center. As expected, the spatial distortion is smaller for higher magnifications. The measurements at 50*μm* HFV slightly differ from the ones at 600*μm*, which may be caused by the fact that the 50 and 10 *μm* HFV tests were done on a different day than the 600 *μm* HFV tests (different electron beam parameters). The spatial distortion at 10*μm* field of view is slightly below the noise level in the measurements, and thus may be neglected for measurements at this length scale. Nevertheless, at each magnification, the reproducibility of the spatial distortion is within the expected accuracy.

**Fig. 16:**
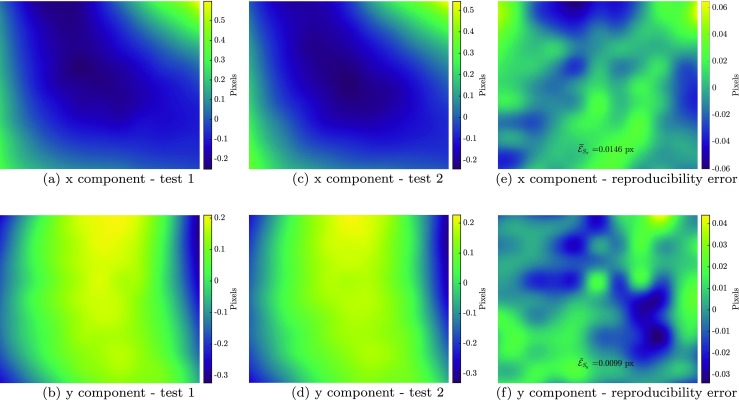
Spatial distortion field measured on 600*μm* field of view for measurement 1 in **a**, **b**, measurement 2 in **c**, **d**. The difference between the two measurements is shown in **e**, **f**

**Fig. 17:**
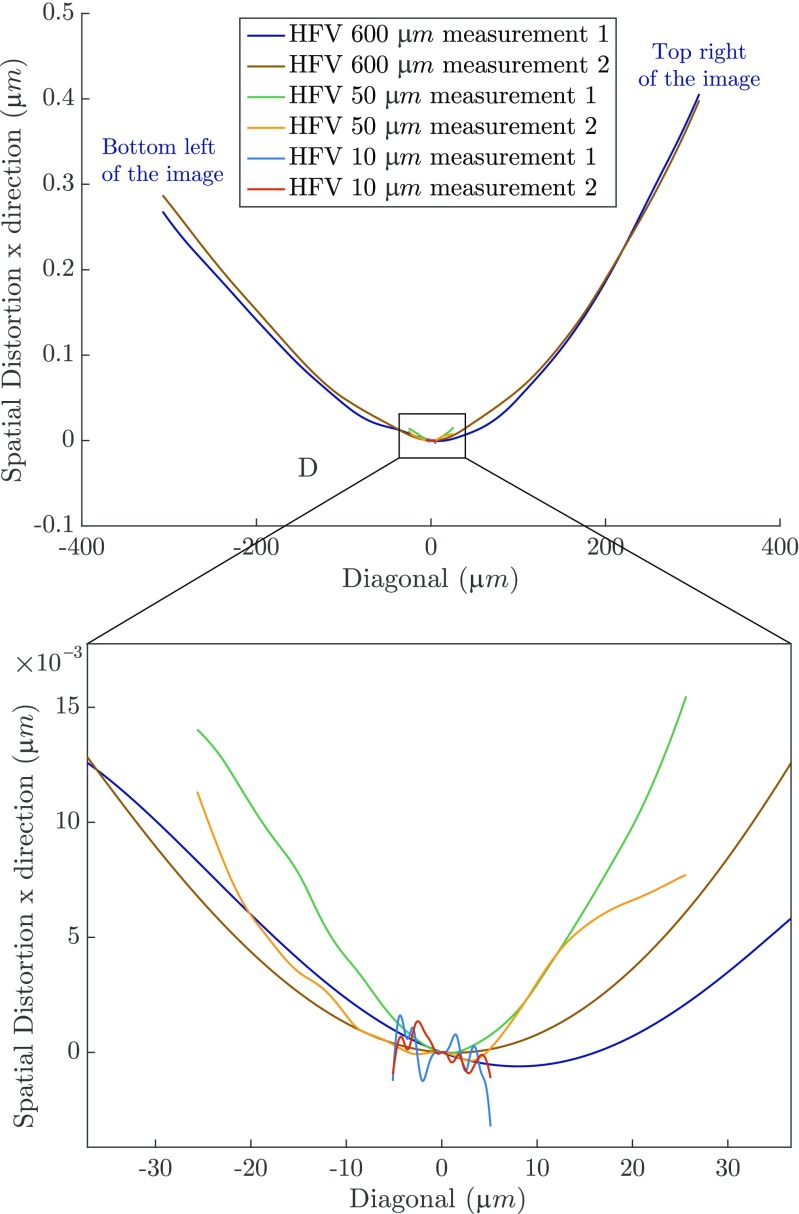
Comparison of spatial distortion measurements at different magnifications, where the x component is plotted as a function of the diagonal (bottom left to top right) of the images, while the bottom graph provides a zoom of the higher magnification measurements

The obtained results for the drift distortion measurements are shown in Fig. 18, where Fig. 18(a) and (b) show the results in pixels while Fig. 18(c) and (d) depict the same results in micrometers. Each curve represents the evolution of drift in time for all eight images in a series. The gaps between segments in each curve represent the dead time between scanning of any two images. Note that although drift distortion is treated as a smooth function in time, also in between the scans, it is plotted only for the scanning duration of each image where actual measurement data exists. It is observed that the drift in pixels is much more pronounced at higher magnifications. The drift measured in the tests at 600*μm* HFV is as small as the accuracy of DIC, i.e. 0.01 px, which is why these noisy measurements are omitted in Fig. 18(c) and (d). Note that Fig. 18(c) and (d), show that the effect of drift distortion on a physical scale, i.e. expressed in *μm*, is independent of the magnification and comparable in rate and direction. This shows that in this particular case, drift distortion is dominated by a physical motion (e.g. due to a motion of the stage with respect to the column). This can be understood from the fact that all the four tests at 50 and 10*μm* field of view were performed in one session, in which the drift distortion apparently occurred mainly in *y* direction having a more or less similar rate.

**Fig. 18:**
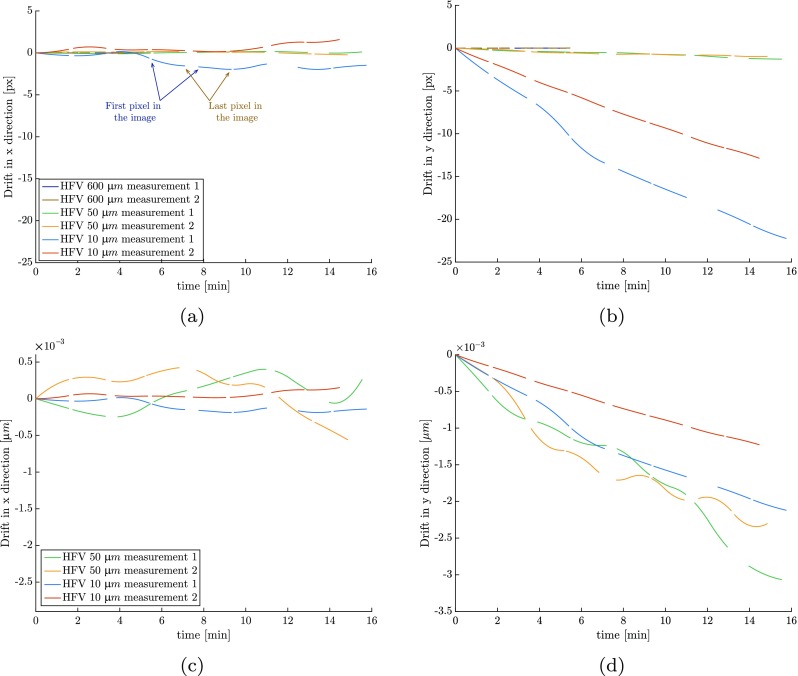
Evolution of drift as a function of time for each image series, plotted in **a**, **b** pixels and **c**, **d** micrometers. Drift distortion is a smooth function in time; however, it is plotted only for the duration of each image

The third type of considered artifact, i.e. line shifts, are the most pronounced and hardest to deal with for the images with 10*μm* field of view. In the second 10*μm* HFV test, the first image is interesting as it contains one line shift. LDIC is used between this first (reference) and the second image in the series, which also contains a scan line shift. Note that LDIC is only used for evaluating the impact of the line shift artifact corrections. For this case, where the mechanical displacement is constant in space (rigid body motion), it is possible to use LDIC as a reference, which means that any variation in the displacement field, found by LDIC, is due to the SEM artifacts. LDIC is performed with 53 px subset and 1 px step size, using VIC-2D™. As the amplitudes of the scan line shifts in *x* and *y* direction are comparable, Fig. 19(a) only shows the displacement field in *x* direction, obtained from LDIC on the second image in the second series of 10*μm* HFV, where two line shifts are visible, both exhibiting negative amplitudes. The line shifts measured with the proposed artifact correction method are depicted for *x* direction in Fig. 19(b), where the left and right sides show the line shift in Image 1 and 2. Note that the line shift in Image 1 has a positive amplitude while LDIC shows a negative amplitude since it cannot distinguish between the artifacts of the two images.

**Fig. 19:**
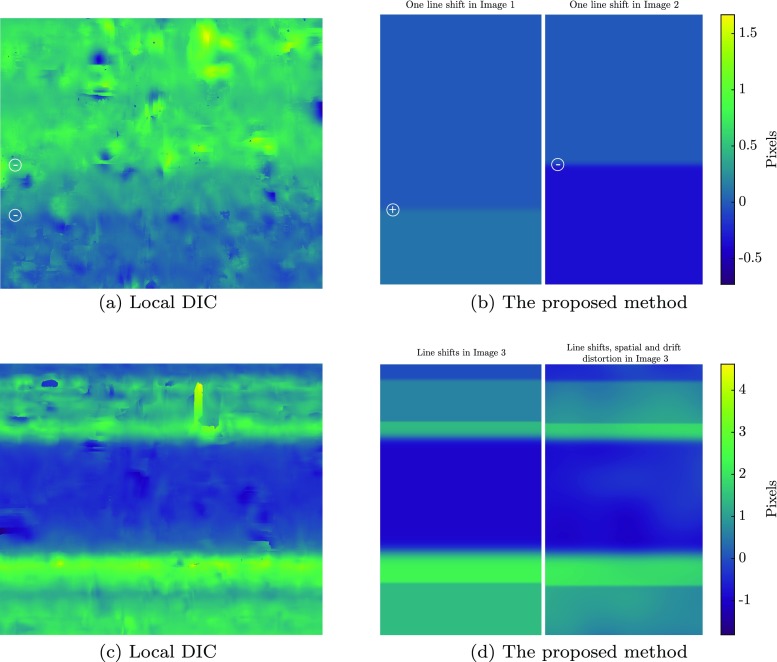
Measurement of line shift artifacts and comparison with local DIC results. **a** Displacement in x direction of LDIC on the second image of test 2 with 10*μm* field of view compared to **b** line shift artifacts measured with the proposed method in image 1 (left) and 2 (right). **c** Displacement in x direction of LDIC on the third image of test 1 with 10*μm* field of view compared to **d** line shift artifacts (left) and the total artifact field (right) measured with the proposed method in the same image. In all the cases here the amplitudes of the scan line shifts in *y* direction are comparable to the amplitudes in *x* direction

One of the images in the first 10*μm* HFV test contains multiple line shifts which is therefore also analyzed by comparison to LDIC. As the amplitudes of the scan line shifts in *x* and *y* direction are comparable, Fig. 19(a) only shows the displacement field in *x* direction from LDIC. Five line shifts are visible in this image, which is beyond the expected limitations of the proposed method [32]. Yet, all five line shifts are successfully identified, see Fig. 19(d). The line shift amplitudes moreover match well with those from LDIC. However, the line shift locations reveal a minor deviation, which is caused by the other distortion fields displacing the line shifts. The LDIC results should, therefore, be compared with the proposed method including all artifact corrections. This is depicted on the right side of Fig. 19(d), which restores the expected match with the line shift locations revealed by the LDIC results. Note the noisy results of LDIC (due to the suboptimal DIC pattern) in comparison with the low level of noise in the artifact measurement by the proposed method.

Figure 20 finally compares the residual fields with and without artifact correction. Figure 20(a) shows the residual field of one of the images with HFV 10*μm* from a conventional GDIC ignoring all artifacts, where the mechanical mapping function has been parametrized with zeroth order polynomials. Figure 20(b) depicts the residual field of the same image from the correlation including the artifact corrections. The difference between the two emphasizes the quality of the measurement of the artifacts and the correction for them. Such an accurate measurement of the different artifact fields is a necessary condition for eliminating the artifact-induced errors in the mechanical deformation measurements from *in-situ* SEM tests. Figure 20(c) and (d) show the same comparison for the 50*μm* field of view, where a zoomed view is included for clarity. Similar results are also obtained for the lowest magnification tests.

**Fig. 20:**
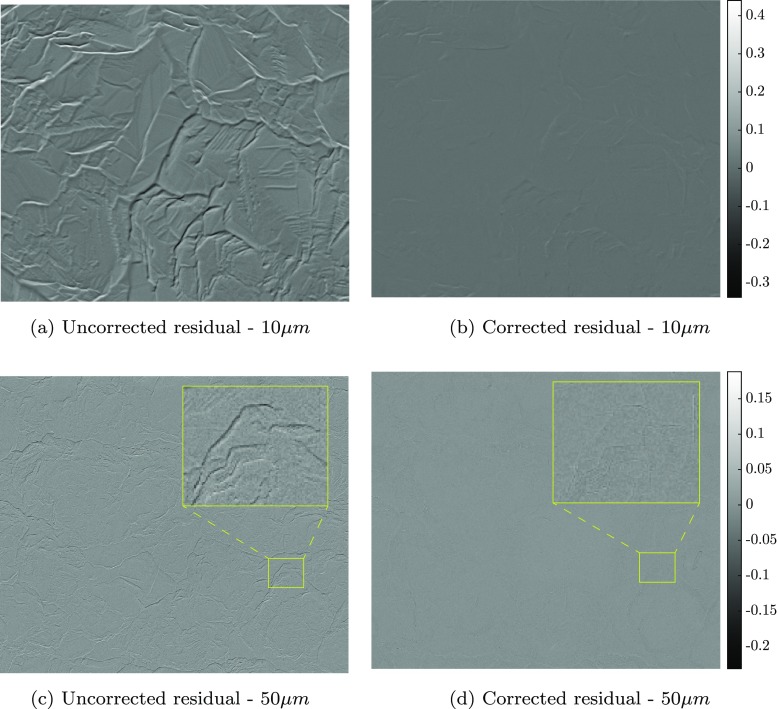
Examples of residual fields of images with 10*μm* horizontal field of view **a** without and **b** with artifact correction, and of images with 50*μm* horizontal field of view **c** without and **d** with artifact correction (zoomed view included for clarity)

## Conclusions

Using high resolution scanning electron microscopy images should enable high displacement resolutions in DIC. However, SEM images contain artifacts which introduce considerable errors in displacement measurements if ignored. SEM artifacts are categorized in three types: spatial distortion, drift distortion and scan line shifts.

The current study proposes a generic unified framework based on IDIC to measure all three types of artifact fields alongside the mechanical deformations, in an integrated manner, to minimize the artifact induced errors in the displacement measurements. To this purpose, the imaging process of SEM is captured through the hierarchical mapping functions that have been inserted in the proposed IDIC framework. Based on these hierarchical mapping functions, the IDIC problem is reformulated. Using the proposed IDIC framework and following the proper imaging and correlation procedures, the artifact fields can be measured separately from the mechanical displacement fields in a simple optimization step. This separation is made possible through the physical characteristics of the individual fields:

(i) spatial distortion is inherently a constant field in time; it is identified during an independent calibration step in which no mechanical deformation occurs (only discrete steps of rigid body motion are applied);
(ii) drift is a continuously evolving, smooth function in time, also during scanning of each image; its image distortion is distinguished from mechanical deformation which is applied in a step-wise manner between the acquisition of every two images, making it constant in each image pair;
(iii) scan line shift artifact fields are random localized distortions with a direction dictated by the underlying scanning process; they occur discretely in time, and are distinguished from the mechanical deformation through image pairs, similar to drift;
(iv) mechanical deformation is considered as an arbitrary complementary field, constant within a given loading step (i.e. constant for each image pair); hence no constraint is enforced on mechanical deformation.

This methodology has been validated with a series of virtual experiments. First, artificially generated images have been deformed both by an evolving mechanical deformation field and by SEM imaging artifact fields. The mechanics and the artifacts of each of these sets of images have been then measured using the framework introduced in this article. First, a less complex case of mechanical deformation and spatial distortion has been studied in a virtual experiment, and analyses on noise levels and the regularization of the artifact fields have been conducted. It has been shown that the error in the mechanical displacement measurement remains acceptable up to a noise level of 5*%* of the image dynamic range. Different regularizations of spatial distortion with global basis functions and drift distortion with both globally and locally supported basis functions, resulted in acceptable error levels (< 0.02 px) in the mechanical displacement measurements, confirming the robustness of the framework in convergence.

Second, a more complex virtual experiment has been carried out by deforming another set of virtually generated images reflecting a challenging localized mechanical deformation and asymmetric spatial distortion field (taken from SEM-DIC measurements in the literature). The artifact and deformation fields have been measured using the current method, which resulted in errors well within the DIC accuracy range.

Third, the same challenging virtual experiments have been repeated using real SEM patterns to study the performance of the proposed methodology under realistic conditions. A regular SEM-DIC pattern provides the same accuracy as a virtual pattern. A sub-optimal multiscale pattern (based on an electro-deposited copper film) reveals somewhat higher errors in the evaluation of the mechanical and distortion fields.

And finally, the proposed method has been validated on different sets of real SEM images at three different magnifications, based on the sub-optimal multiscale pattern, to assess the accuracy with which the artifacts can be measured. The reproducibility of the results of spatial distortion and drift distortion, the overlap with measurements at different magnifications, and low image residuals show the accuracy of the measurements. The comparison of the line shift artifact measurements with LDIC results reveals the accuracy of the line shift artifact measurements even when five line shifts occur in one image, which is beyond the expected limitation of the proposed method. Finally, the significant improvement of the residual fields by including the artifact corrections confirms the high accuracy of the artifact corrections performed using the current method.

The proposed method is unique in the following:

(i) it deals with all three types of SEM artifacts (line shits, drift and spatial distortion) in a unified and systematic way,
(ii) the SEM imaging process is taken into account through a set of hierarchical mapping functions (the general framework can be easily extended to any imaging system),
(iii) all artifact and mechanical deformation fields are captured properly in only two correlation steps (spatial distortion calibration and mechanical test phase),
(iv) the acquired data (SEM images) are used most efficiently by avoiding any integration of images,
(v) the drift distortion is measured/corrected directly for all the duration of the test including the reference image, without any extrapolation of data.

## Figures and Tables

**Fig. 21:**
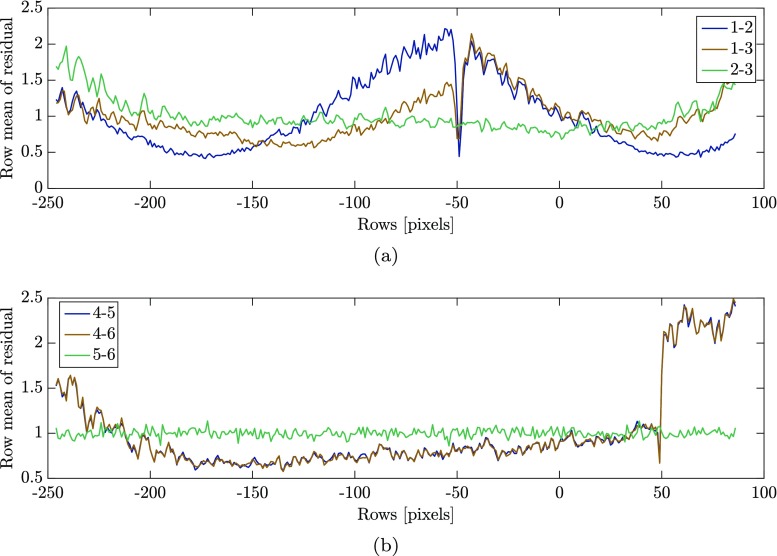
Row mean of the residuals of pre-correlations as a function of the row number for the virtual images of the calibration phase. Triplets of **a** 1, 2 and 3 revealing a line shift in image 1, approximately at *y* = − 50 px and **b** 4, 5 and 6 revealing a line shift in image 4, approximately at *y* = 50 px. Note that the residual fields are available only in the region of interest

## Appendix A: Alternative image gradient arrangement

It can be easily shown that by rearranging the image gradient, as found in Ref. [37] for the case of conventional GDIC, equations (21) to (23) can be rewritten as:

This arrangement is more convenient for implementation, mainly because there is no need for interpolation in the image gradient calculation.

## Appendix B: Initial guess for correlations

In order to establish convergence of the correlations, a systematic procedure is adopted. It is proposed to follow these few steps of correlations by gradually adding some of the dofs and removing blurring from images. Each step improves the initial guess for the next one, so that starting with the most trivial initial guess (zero), the correlations converge robustly and monotonically.

The randomly occurring line shifts need to be detected and an adequate initial guess of their positions is needed. Following [32], a pre-correlation step is performed in which the approximate location of the line shifts is identified. This is done by performing a conventional GDIC analysis between image pairs, ignoring the existence of any line shifts and using merely first order polynomials for basis functions. This pre-correlation is the most simple GDIC correlation. The residual fields of the pre-correlations reflect the line shifts. Plotting the row mean of the residual fields as a function of the row number of the images reveals existing line shifts, which gives a good initial guess for their positions, cf. Fig. 21. Comparing these positions for three correlations among three images indicates which line shift belongs to which image. Figure 21 depicts an example of 6 pre-correlations, of a set of six SEM images of the calibration phase of the virtual experiment. Figure 21 shows the pre-correlations between images 1, 2 and 3. There is a high gradient in the curves related to the pre-correlations involving image one at approximately *y* = − 50 px reflecting a line shift artifact in image 1 and in this position. Likewise, Fig. 21b reveals a line shift at approximately *y* = 50 px in image four. It is also helpful to use smoothed derivatives of these curves in which dominant peaks indicate line shifts in the images. By this means the procedure is even automated. Note that in cases in which recognition of line shifts becomes more difficult, e.g. due to a local lack of DIC pattern or the presence of several line shifts close to one another, LDIC can provide a useful indicator. The width of each line shift is initially constrained to a large value, e.g. 20 px, to first correlate the position as well as the line shift amplitudes along with all the other deformation and distortion fields, after which a final correlation step including also the dofs for the line shift width is performed. Once proper initial guesses for line shift artifact positions are attained, the very first correlation step is performed on images that are blurred by a Gaussian filter with standard deviation of 10 px and a window size of 41 px. After obtaining proper initial guesses for the displacement and artifact fields, the second correlation step is performed on the original images (not blurred). In the first two correlation steps, the dofs characterizing the width of the line shifts are deactivated, as mentioned above. The next step is basically a repetition of the previous step including the dofs of the width of all the line shifts. Up to this point the reference image is chosen to be the first back-deformed image ($\tilde {g}_{1}$), as in equation (11). The final (principal) step is correlating the original (not blurred) images with all the dofs of mechanics and artifacts, and the reference image defined as the average of all back-deformed images, as in equation (29). Since the correlation with the general definition of the reference image typically requires more iterations, the extended definition for the reference image is introduced only when the solution is almost approached. This optimizes the total number of iterations. The steps to be taken can be summarized as:

- perform pre-correlation on image pairs with first order polynomials,
- plot the row mean of the residual field of each three pre-correlations and obtain the initial guess for the position of line shifts,
- perform a correlation step on blurred images to obtain a good initial guess for all dofs,
- perform the second correlation step on the original (not blurred) images,
- repeat the final step with the reference image defined as the average of all back-deformed images.

## Footnotes

1 Sutton et al.’s method [11] consists of the following steps. Local DIC is performed on image pairs to get drift distortion disparity maps. B-spline surfaces are fitted to these maps. For each pixel separately, the drift velocity in time is found at certain points in time. The drift velocity is fitted with a B-spline function in time. The fitted drift velocity is integrated to identify drift distortion in time. For all pixels, a B-spline surface is fitted to provide a functional for the drift distortion field for one image of each pair. These disparity maps are corrected for drift distortion. A B-spline surface is fitted to these disparity maps resulting in a functional for spatial distortion. The (drift-corrected) disparity maps are corrected for spatial distortion. The whole process is repeated until both drift and spatial distortion are corrected.
